# Progress and Challenges of Vanadium Oxide Cathodes for Rechargeable Magnesium Batteries

**DOI:** 10.3390/molecules29143349

**Published:** 2024-07-17

**Authors:** Elena G. Tolstopyatova, Yulia D. Salnikova, Rudolf Holze, Veniamin V. Kondratiev

**Affiliations:** 1 Institute of Chemistry, Saint Petersburg State University, 7/9 Universitetskaya Nab., 199034 Saint Petersburg, Russia; 2 State Key Laboratory of Materials-Oriented Chemical Engineering, School of Energy Science and Engineering, Nanjing Tech University, Nanjing 211816, China; 3 Chemnitz University of Technology, 09107 Chemnitz, Germany; 4 Confucius Energy Storage Lab, School of Energy and Environment, Southeast University, Nanjing 210096, China

**Keywords:** magnesium ion battery, cathode materials, electrochemical performance

## Abstract

Among the challenges related to rechargeable magnesium batteries (RMBs) still not resolved are positive electrode materials with sufficient charge storage and rate capability as well as stability and raw material resources. Out of the materials proposed and studied so far, vanadium oxides stand out for these requirements, but significant further improvements are expected and required. They will be based on new materials and an improved understanding of their mode of operation. This report provides a critical review focused on this material, which is embedded in a brief overview on the general subject. It starts with the main strategic ways to design layered vanadium oxides cathodes for RMBs. Taking these examples in more detail, the typical issues and challenges often missed in broader overviews and reviews are discussed. In particular, issues related to the electrochemistry of intercalation processes in layered vanadium oxides; advantageous strategies for the development of vanadium oxide composite cathodes; their mechanism in aqueous, “wet”, and dry non-aqueous aprotic systems; and the possibility of co-intercalation processes involving protons and magnesium ions are considered. The perspectives for future development of vanadium oxide-based cathode materials are finally discussed and summarized.

## 1. Introduction

Magnesium ion batteries are among the promising power sources that can provide high energy density combined with increased environmental and operational safety at lower device costs mostly because of the high magnesium content in the Earth’s crust [1,2,3,4,5,6]. The Mg/Mg^2+^ system provides metallic anodes offering a high theoretical specific capacity (3834 mAh·cm^−3^), which is higher than that of metallic lithium (2061 mAh·cm^−3^). It also provides an attractively low electrode potential (−2.37 V) and forms metallic deposits without dendrites [1,4,5,6].

Due to these advantages, rechargeable magnesium ion batteries have attracted considerable and growing research interest in the field of post-lithium secondary batteries [7]. For comparison, Table 1 collects pertinent data on relevant negative electrode materials.

Systems with both non-aqueous and aqueous electrolytes are considered, in addition to the extended window of electrochemical stability available with water-in-salt electrolytes [8] has been discussed and extended into a quasi-solid state concept [9]. A first pouch cell demonstration has been reported [10].

Challenges for developing a practical magnesium full cell are related to all components, including in particular Mg^2+^-containing electrolytes that would remain electrochemically stable with both a Mg anode and cathodes. Nonaqueous rechargeable magnesium batteries also suffer from the complicated and moisture-sensitive electrolyte chemistry at magnesium electrode. Practical realization of a RMB is, in particular, handicapped by the absence of high-performance electrode materials due to the intrinsically slow Mg^2+^-ion diffusion in solids.

**Table 1:** Selected data of relevant negative electrode materials for metal ion batteries [11].

Element	Atomic Mass	*E*_0_, SHE/V	Gravimetric Capacity/mAh·g^−1^	Volumetric Capacity/mAh·cm^−3^
Li	6.94	−3.040	3860	2061
Na	23.0	−2.713	1165	1129
K	39.1	−2.924	685	610
Mg	24.31	−2.356	2206	3834
Ca	40.08	−2.840	1337	2072
Zn	65.41	−0.763	820	5855
Al	26.98	−1.676	2980	8046

Critical characteristics, such as specific capacity, rate capability, and cycling stability of RMBs, are strongly affected by the intrinsic electrochemical properties of the cathode, i.e., the positive electrode (following an earlier recommendation by Huggins [12,13]), and, in particular, the materials they are made of. There is continuous progress in the improvements of their properties.

Several earlier reviews [14,15,16,17] as well as selective overviews [10,18,19,20,21,22,23] on RMBs are available. They address cathode-related issues like electrode reaction kinetics particularly relevant for rate capability and mass utilization [24] and detrimental effects of particular properties attributed to Mg^2+^-ions [25], highlight potential advantages of layered compounds [26], and provide mostly brief considerations and discussions of aspects concerning different intercalation-type cathode materials (like transition metal oxides, phosphates, chalcogenides, and Prussian blue analogues), but they are usually very short on considerations of their properties and mostly without an in-depth analysis of the reasons for the differences in functional properties (like specific capacity and power) and mechanism of intercalation processes regarding the latter class of materials. A focal issue is the high charge density of the magnesium ion with associated problems of strong interactions with a host material used in the positive electrode and associated slow diffusion. This also affects interfacial processes at the electrode/solution interface, and possibly even the electrolyte solution.

The number of investigations on different cathode materials for RMBs is rapidly increasing every year. Several reviews are published each year summarizing experimental progress on various cathode materials suitable for RMBs (transition metal oxides, sulfides, selenides, and other layered materials) [10,19,26,27,28,29,30,31,32,33,34,35,36,37,38,39,40]. However, given the large number of material classes and research areas, they are discussed too superficially, without more detailed consideration of the existing challenges.

Some more specialized reviews discuss and analyze reaction mechanisms in cathodes structure–kinetics correlations, strategies for improving the electrode kinetics [24], the role of the electrolyte composition in the intercalation processes of Mg^2+^-ions [41], cathode materials and electrolytes for aqueous magnesium batteries [42], nanostructured cathode materials [43], and employing graphene and graphene-based materials to improve cathode materials [44].

Some reviews on RMBs have been dedicated to vanadium oxides solely. The structural characteristics and electrochemical performance of vanadium-based materials as RMB cathodes are discussed in [6,45,46]. The role of structural water molecules in vanadium oxide was analyzed in [47], the developments in application of vanadium oxide bronzes in metal ion batteries were discussed in [48], and the intercalation mechanisms in vanadium oxide were discussed in [49,50].

In this review, we will focus on the recent investigations of one of the promising and widely studied groups of cathode materials—layered vanadium oxides, including a systematic discussion of the achievements in their development and their possible improvement, the existing opinions on the mechanisms of charge–discharge processes in aqueous, “wet”, and dry non-aqueous electrolytes, and strategies to optimize functional properties of these cathode materials. We believe that this specialized and detailed review will help researchers to optimize the functional properties of these cathode materials.

## 2. Vanadium Oxide-Based Cathodes

Why did we select vanadium oxides as cathode materials for RMBs for this review?

Although the radius of Mg^2+^-ions (0.72 Å) is similar to that of Li^+^-ions (0.76 Å), the intercalation of Mg^2+^-ions into the same host materials is more difficult due to the difference in charge. The large charge/radius ratio of Mg^2+^-ions results in their strong electrostatic interactions with cathode materials, making intercalation and diffusion processes sluggish. As a result, most cathode materials suitable for RMBs exhibit a low degree of magnesiation, large voltage hysteresis, and low rate capabilities. Therefore, it is important to develop high-performance cathode materials for RMBs.

Vanadium-based materials have high theoretical capacity and energy density resulting from the multiple valence states of vanadium. Many vanadium-based materials have a layered structure, and the interlayer can be enlarged and adjusted to favor the intercalation and diffusion of Mg^2+^-ions into the cathode material.

Among vanadium-based materials, vanadium oxides are the most common and have numerous advantages for multivalent metal ion batteries. Vanadium oxides are cheap and abundant. Vanadium oxides with different structures have a variety of valence states (V^+3^/V^+4^/V^+5^) and can be easily synthesized by a variety of methods. Vanadium oxide polymorphs with different lattice symmetries have different electronic properties and thus different metal ion insertion thermodynamics and kinetics. Different synthetic strategies offer possibilities to synthesize different polymorphic structures and to fit the obtained layered structures to different interlayer distances and to weaken (i.e., screen) the strong electrostatic interaction of Mg^2+^-ions with the host lattice.

Among other cathode materials, the vanadium pentoxide family has been considered promising for RMBs due to the high theoretical capacity of V_2_O_5_ (294 mAh·g^−1^, considering 1 mol of Mg^2+^ per mol of V_2_O_5_) and high working voltage of ≈2.4 V, resulting in a high specific energy (>600 Wh·kg^−1^) [6,51,52,53]. Orthorhombic V_2_O_5_ was the first of the vanadium oxides to be investigated for Mg^2+^ intercalation.

In recent years, researchers have focused on other types of vanadium oxides like V_2_O_5_·*n*H_2_O, M_x_V_2_O_5_·*n*H_2_O (with M: intercalated foreign metal ion) [54], VO_2_ [55,56], and others, and also specially designed mixed-valence composites, such as V_3_O_7_/VO_2_ [57].

**Figure 1:**
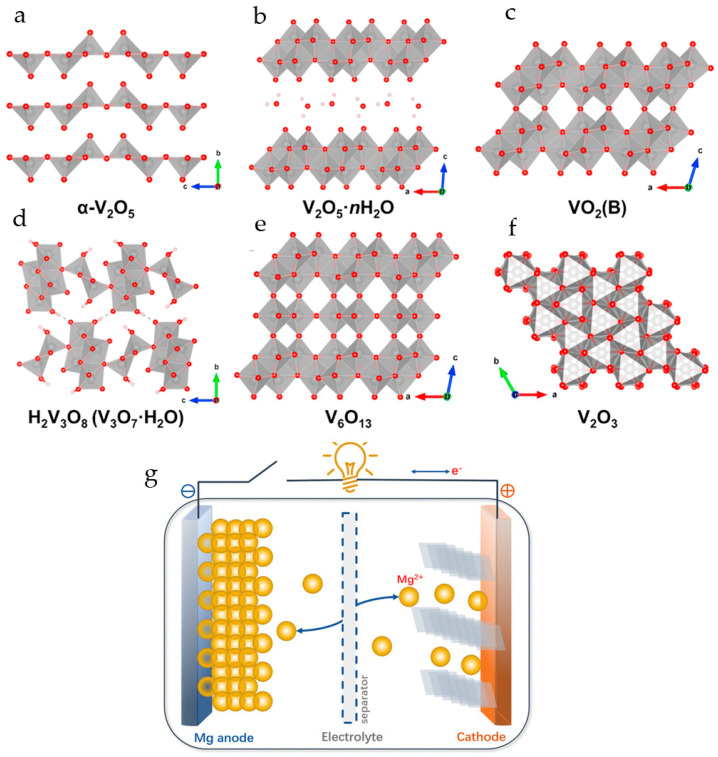
Crystal structures of vanadium oxides: (**a**) α-V_2_O_5_, (**b**) V_2_O_5_·*n*H_2_O, (**c**) VO_2_(B), (**d**) H_2_V_3_O_8_ (V_3_O_7_·H_2_O), (**e**) V_6_O_13_, (**f**) V_2_O_3_. (Reprinted with permission from [45]. Copyright 2023, Elsevier). (**g**) The working principle of the RMB. (Reprinted with permission from [21]. Copyright 2020, John Wiley and Sons).

In the thermodynamically most stable α-phase, V_2_O_5_ has a layered structure consisting of alternating edge- and corner-sharing distorted VO_5_ trigonal-bipyramidal coordination polyhedrons stacked along the *c*-axis with an interlayer spacing of 4.37 Å (Figure 1a). Vanadium oxide gels (V_2_O_5_·*n*H_2_O) have bilayer crystal structures comprised of layered square-pyramidal VO_5_ polyhedra in which V^5+^ is coordinated with five oxygen atoms. Water molecules are bound between the layers of V_2_O_5_ (Figure 1b). The reported water content *n* in V_2_O_5_·*n*H_2_O is usually about 1.6–2.0 moles per mole of V_2_O_5_ [58]. The layered structure provides channels for ion insertion and de-insertion (Figure 1b).

Brookite-phase vanadium dioxide VO_2_(B) (Figure 1c) has a stable open framework. The crystal structure of VO_2_(B) consists of corner- and edge-sharing VO_6_ octahedra that provide three plausible migration channels along the *c*- and *b*-axes. It has a monoclinic symmetry with a space group of *C2/m*. Because the *b*-axis is the shortest unit cell dimension, crystals grow predominantly along the *b*-axis, forming one-dimensional morphologies such as nanowires, nanorods, and nanobelts. VO_2_(B) is a promising candidate for RMBs due to its high theoretical capacity of 323 mAh·g^−1^ per one electron transfer for the V^4+^/V^3+^ redox coupe [56].

Hydrated vanadium oxide (H_2_V_3_O_8_, V_3_O_7_·H_2_O, HVO) (Figure 1d) has an orthorhombic crystal structure and comprises V_3_O_8_ layers, consisting of edge- and corner-shared VO_5_ square pyramids and VO_6_ octahedra. In contrast to van der Waals forces in V_2_O_5_, vanadium oxide layers in HVO are linked by hydrogen bonds, which decrease the volume expansion/contraction during cycling.

The crystal structure of V_6_O_13_ (Figure 1e) is composed of corner- and edge-sharing distorted VO_6_ octahedra, which form alternating single and double vanadium oxide layers connected by shared corners. V_6_O_13_ has high electrical conductivity at room temperature and has a high theoretical capacity of 417 mAh·g^−1^ when high-valent vanadium (V^5+^/V^4+^) is all reduced to V^3+^.

The vanadium trioxide V_2_O_3_ (Figure 1f) possesses a tunnel-like 3D structure, which determines its good ion intercalation performance. The theoretical capacity of V_2_O_3_ is 357.5 mAh g^−1^ and 715.1 mAh g^−1^ for one- and two-electron redox processes.

Consideration of this class of insertion-type compounds allows to highlight the main strategic ways to design layered metal oxide cathodes and to consider, on these examples, the typical issues and mechanistic details often missing within broader overviews and reviews.

In particular, these issues should be focused on (i) the electrochemistry of intercalation processes in layered oxides (vanadium oxide V_2_O_5_ and its modifications), their mechanism in aqueous, wet, and dry non-aqueous aprotic systems (PC, AN), the possibility of co-intercalation processes involving protons and magnesium ions; and (ii) the dependence of electrochemical properties on the modification of the interlayer space due to the inclusion of foreign metal ions, organic molecules (polymeric and others), and water molecules.

The basis for the selection of a suitable inorganic host material in metal ion battery technology is the reversible electrochemical intercalation processes associated with electrochemical redox processes with the recharging of metal ions and the accompanying reversible processes of insertion/removal of electrolyte cations into/from the host electrode material (Figure 1g). The driving force of these processes is the gradient of electrochemical potentials realized in the host materials. The three main factors affecting the intercalation and diffusion of metal ions are (i) steric factors, depending on the geometric size of intercalated metal ions and crystal structures providing the interstitial voids along ion hopping trajectories, (ii) desolvation of ions at injection, and (iii) electrostatic and other interactions of intercalated ions with the host materials.

Several innovative approaches have been proposed for layered oxide cathodes to successfully overcome all these challenges in the case of Mg^2+^-intercalation. Among them is the strategy based on a significant increase in the interlayer spacing in layered materials by the synthesis of layered materials pre-intercalated/doped with foreign metal ions or organic molecules, which could facilitate Mg^2+^-intercalation and diffusion in the host material. Another important feature of the increased interlayer spacing is the possible intercalation of solvated magnesium ions, avoiding the sluggish desolvation process at the interface.

Additional attention is given to the retention of water molecules in the host structures and the co-insertion of water molecules in the case of aqueous or wet non-aqueous electrolytes. The degree of reversible Mg^2+^-insertion strongly depends on the “lubrication” of the layered channels. The selected strategic approaches are usually combined with more common measures, such as nanostructuring of materials, which results in shortening the solid state diffusion path for highly polarized Mg^2+^ in the host and increasing the surface area of the active grains. In the following, we discuss these issues in more detail, based on experimental examples from published original works.

### 2.1. Orthorhombic Vanadium Pentoxide V_2_O_5_

Orthorhombic vanadium pentoxide (α-V_2_O_5_) was the first compound in the family of vanadium oxides to be proposed for use as cathode in RMBs. Early works showed that its typical layered crystal structure with weak van der Waals forces is suitable for tuning the interlayer space, making it accessible for intercalation of Mg^2+^-ions [29,59,60,61].

The electrochemical intercalation of Mg^2+^-ions into orthorhombic V_2_O_5_ in a solid state RMB composed of a metallic Mg anode, a solid Mg-montmorillonite electrolyte, and a V_2_O_5_ cathode has been confirmed [59]. The quantitative estimation of magnesium intercalation was reported [60], where the authors reported that 0.66 mol of Mg can be intercalated into 1 mol of V_2_O_5_ by chemical intercalation in the dibutylmagnesium/heptane solution, which corresponds to a high specific capacity of 194 mAh·g^−1^. This value is consistent with the capacity determined electrochemically in a 1 M Mg(ClO_4_)_2_/THF electrolyte.

A stronger indication that V_2_O_5_ is a potential cathode material for RMBs was the first publication on the ability of α-V_2_O_5_ to reversibly electrochemically intercalate Mg^2+^ in 1 M Mg(ClO_4_)_2_/AN electrolytes with water added to AN [61]. It was shown that the insertion of Mg^2+^ into V_2_O_5_ depends on the ratio between the amounts of H_2_O and Mg^2+^ and on the absolute amount of H_2_O in the electrolyte solution. The preferential solvation of Mg^2+^-ions by water molecules facilitated the insertion process. The highest capacities of up to 170 Ah·kg^−1^ were obtained in acetonitrile solutions containing 1 M Mg(ClO_4_)_2_ + 1 M H_2_O; however, the electrode had low cycling stability—only about 50 Ah·kg^−1^ was retained after 20 cycles.

To optimize the diffusion pathways of Mg^2+^ in V_2_O_5_ during the charge–discharge, orthorhombic V_2_O_5_ with particle size in the range of 20 to 50 nm obtained via a combustion flame-chemical vapor condensation process was suggested [62]. The reversible capacity of the V_2_O_5_ in 0.5 M Mg(ClO_4_)_2_/PC was 180 mAh·g^−1^ at a current density 7.6 mA·g^−1^.

During the discharge process, Mg^2+^-ions are gradually inserted into the interlayer of V_2_O_5_ and become coordinated with the VO_5_ pyramids, and the oxidation state of vanadium decreases from +5 to +4. The V^4+^/V^5+^ redox pair endows V_2_O_5_ with a high potential of 2.66 V vs. Mg/Mg^2+^. The overall reaction of the electrochemical insertion of Mg^2+^ ions into V_2_O_5_ as a RMB cathode material can be written as

(1) $$V_{2}O_{5}{+x\;\mathrm{Mg}}^{2+}{+2x\;e}^{-}\;\underset{\;}{⇄}\;{\mathrm{Mg}}_{x}V_{2}O_{5}$$

V_2_O_5_ has a high theoretical specific capacity of 294 mAh·g^−1^ when 1 mol of Mg^2+^ ion is inserted (x = 1) [28].

V_2_O_5_ forms metastable polymorphs (β-, γ-, δ-, ε-, and ζ-V_2_O_5_) in addition to the thermodynamically stable phase (α-V_2_O_5_). Some of them have been the subject of theoretical and experimental studies concerning RMBs.

First-principles calculations were performed to investigate Mg intercalation in the α- and δ-polymorphs of V_2_O_5_ [63]. It was supposed that the conversion of fully demagnesiated stable α-V_2_O_5_ into a two-phase structure consisting of fully magnesiated δ-V_2_O_5_ and fully demagnesiated α-V_2_O_5_ phases during the discharge process results in slow diffusion kinetics in V_2_O_5_ electrodes. It was shown that since the calculated α-phase migration barriers indicate poor Mg mobility, reversible Mg intercalation is only possible at very low rates and in small particles, and the δ-V_2_O_5_ polymorph exhibits superior Mg mobility, provided that the δ-V_2_O_5_ host structure can remain stable or metastable over a wide Mg concentration range. The first-principles calculations have also shown that the Mg^2+^-ion diffusion barrier in δ-V_2_O_5_ (~0.6–0.8 eV) is lower than that in α-V_2_O_5_ (~0.975–1.1 eV) [64].

The DFT calculations have also shown that V_2_O_5_ undergoes a structural transformation from the α-phase to the ε-phase (ε-Mg_0.5_V_2_O_5_) and δ-phase (δ-MgV_2_O_5_) as the concentration of intercalated Mg^2+^-ions is increased [65].

The DTF calculations have shown a significant reduction in barriers for Mg diffusion in the β-V_2_O_5_ phase (0.65 eV) compared to α-V_2_O_5_ (>1 eV), leading to possibly fast charge–discharge rates of β-V_2_O_5_ as a cathode material for Mg^2+^-ion batteries [66]. β-V_2_O_5_ synthesized at high pressure demonstrated large voltage hysteresis in a dry (<20 ppm of H_2_O) 0.1 M Mg(ClO_4_)_2_/AN electrolyte and a maximum discharge capacity of 361 mAh·g^−1^ in GCD tests at C/25 [67].

The migration barriers for Mg^2+^ in ζ-V_2_O_5_ calculated by DFT were 0.62–0.86 eV [68], also suggesting its applicability in RMB cathodes. The metastable ζ-V_2_O_5_ obtained by topochemical extraction of the Ag^+^ ion from ζ-Ag_0.33_V_2_O_5_ also demonstrated large voltage hysteresis in Mg(TFSI)_2_/AN electrolyte, and displayed a specific capacity of 90 mAh g^−1^ with insertion of up to 0.33 Mg^2+^ per formula unit [69]. Nanosized ζ-V_2_O_5_ had a discharge capacity of 130 mAh·g^−1^ and lower voltage hysteresis [70].

The experimental investigations also indicated possible multi-stage transformation during the Mg^2+^-intercalation. Mg ion intercalation into nanoscale films of V_2_O_5_ deposited on Pt was studied [71]. It was shown that highly reversible Mg insertion/de-insertion is possible within V_2_O_5_ thin films. In the 0.5 M Mg(ClO_4_)_2_/AN electrolyte, the V_2_O_5_ thin film was cycled over a potential range of 2.2–3.0 V vs. Mg/Mg^2+^, and had an initial capacity of 180 mAh·g^−1^ at a 0.15 mV s^−1^ scan rate, the capacity retention after 36 cycles was ~83%. The electrodes had 100% coulombic efficiency. The differential capacity plots (d*Q*/d*V* − *V*) obtained for low current density galvanostatic cycling revealed four different Mg^2+^-insertion stages or processes with different thermodynamic and kinetic characteristics.

In order to check the inherent ability of α-V_2_O_5_ to intercalate Mg^2+^, electrochemical tests were conducted in an ionic liquid electrolyte in [72]. The α-V_2_O_5_ cathode prepared from commercial vanadium oxide powder had a low reversible capacity of only 16 mAh·g^−1^ at 25 °C, which increased to 295 mAh·g^−1^ (at C/5) at 110 °C. This capacity corresponds to reversible intercalation of 1 mol Mg^2+^ per unit formula. After 1 cycle at 110 °C, the electrochemical activity of α-V_2_O_5_ at room temperature was significantly enhanced (specific capacity of about 95 mAh·g^−1^ at C/5). The composition of the cathode after the thermal activation was Mg_0.2_V_2_O_5_; accordingly, the activation resulted in an expanded interlayer spacing with Mg-pillaring, facilitating fast Mg^2+^-migration. The reversible intercalation of Mg^2+^ into α-V_2_O_5_ was also observed during the characterization of the material composition, crystal structure, and redox state.

Thin films of orthorhombic V_2_O_5_ were grown on fluorine-doped tin oxide (FTO) glass electrodes using AACVD, and studied in aqueous solutions of MgCl_2_ [73]. The material showed a specific discharge capacity as high as 427 mAh·g^−1^ at a high current density of 5.9 A·g^−1^ with 82% capacity retention after 2000 cycles. At a current density of 2.4 A·g^−1^, the specific capacity was 170 mAh·g^−1^, and when the current was returned to 5.9 A·g^−1^, the cathode delivered 425 mAh·g^−1^, corresponding to a capacity retention of 99%. The increase in the specific discharge capacity with increasing specific current was explained by the fast diffusion kinetics of Mg^2+^ in the metal oxide framework.

Several recent studies of V_2_O_5_ nanoclusters have shown that the use of vanadium-based nanoscale materials can improve the diffusion of Mg^2+^-ions and provide high reversibility of intercalation processes. Highly dispersed vanadium oxide nanoclusters supported on porous carbon frameworks were synthesized using the ambient hydrolysis deposition method in order to improve the electrical conductivity of V_2_O_5_ [74]. The composite with 45 wt.% V_2_O_5_ had a surface area and pore volume of 593 m^2^·g^−1^ and 2.8 cm^3^·g^−1^, respectively. Reversible intercalation of Mg^2+^ with an initial capacity of 350 mAh·g^−1^ (per V_2_O_5_) or 180 mAh·g^−1^ (per composite weight) at 40 mA·g^−1^ within the voltage range of 0.5–2.8 V was observed in 0.2 M [Mg2(µ-Cl)_2_(DME)_4_][AlCl_4_]_2_ in DME. During the subsequent five cycles, the capacity of the composite electrode decreased to 160 mAh·g^−1^ (per V_2_O_5_) or 90 mAh·g^−1^ (per composite).

### 2.2. Nanostructured Vanadium Oxides

The findings confirming slow diffusion kinetics in V_2_O_5_ and low capacities pointed at the necessity of modifying the structure of V_2_O_5_ for Mg battery electrode applications.

In most papers it was noted that strong electrostatic interactions between divalent Mg^2+^-ions and lattice oxygen cause slow diffusion of Mg^2+^ in α-V_2_O_5_, and this is one of the main reasons for the unsatisfactory magnesium storage properties. Nanostructuring can increase the active surface area and reduce the diffusion distance to improve the electrochemical performance of α-V_2_O_5_. Various nanostructured vanadium oxide cathode materials with superior electrochemical performance were synthesized.

**Figure 2:**
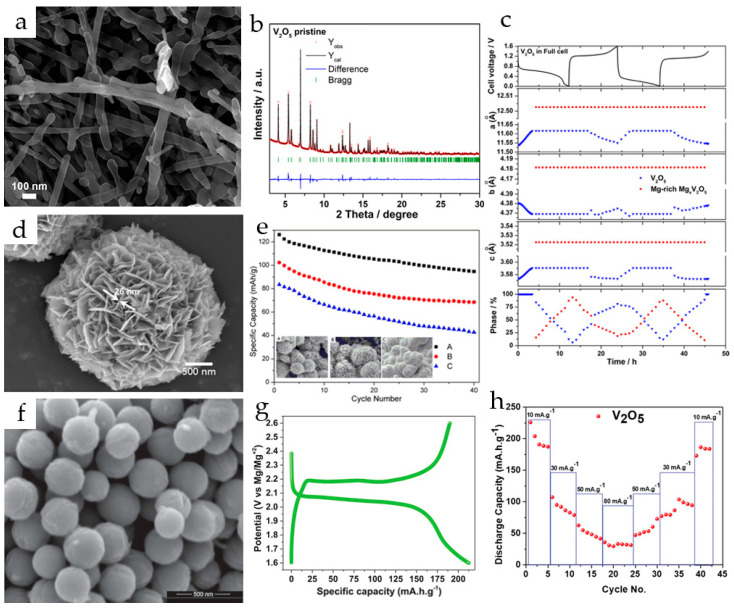
(**a**) SEM image of V_2_O_5_ nanowires, (**b**) synchrotron diffraction pattern of V_2_O_5_ nanowires, (**c**) structural parameters and phase ratios from diffraction patterns with Rietveld refinement during the first two charge–discharge cycles for V_2_O_5_. (Reprinted with permission from [75]. Copyright 2019, American Chemical Society.) (**d**) SEM image of V_2_O_5_ microspheres, (**e**) cyclic performance of V_2_O_5_ microspheres with the diameters of 3 (A), 7 (B), and 15 (C) μm. (Reprinted with permission from [76]. Copyright 2019, Springer). (**f**) SEM (scale 500 nm) image of V_2_O_5_ spheres, (**g**) first galvanostatic profile of V_2_O_5_ spheres at 10 mA·g^−1^, (**h**) rate performance of V_2_O_5_ spheres. (Reprinted with permission from [77]. Copyright 2020, John Wiley and Sons).

Hydrothermally synthesized V_2_O_5_ nanowires (Figure 2a) were used as a cathode in a magnesium ion system with 1 M Mg(ClO_4_)_2_/AN as an electrolyte and Mg_x_Mo_6_S_8_ (x ≈ 2) as an anode [75]. V_2_O_5_ nanowires provided an initial discharge/charge capacity of 103/110 mAh·g^−1^ and a maximum discharge capacity of 130 mAh·g^−1^ in the sixth cycle at a C/20 rate in a full cell.

The synchrotron diffraction pattern of the pristine V_2_O_5_ nanowires (Figure 2b) showed a high degree of crystallinity in the material. All reflections were indexed in the orthorhombic α-V_2_O_5_ structure model with space group Pmn21 and lattice parameters *a* = 11.511 Å, *b* = 4.373 Å and *c* = 3.565 Å. The mechanism of Mg^2+^ insertion and extraction in V_2_O_5_ during the first two cycles was studied by *in operando* synchrotron diffraction (Figure 2c). It was shown that during the first discharge, the V_2_O_5_ phase accommodates Mg^2+^-ions via a solid solution mechanism up to the stoichiometry of Mg_0.14_V_2_O_5_, while the lattice parameters *a* and *c* increased and *b* decreased. During further magnesium uptake, a decrease in the amount of Mg_0.14_V_2_O_5_ phase and an increase in the Mg_0.6_V_2_O_5_ phase with constant cell parameters for both phases were observed; at the end of the first discharge, the phase ratio of Mg_0.14_V_2_O_5_:Mg_0.6_V_2_O_5_ was 13:87. In the second cycle, the material showed almost the same behavior during discharge, and at the end of the second charge V_2_O_5_ returned to its original structure.

Flower-like V_2_O_5_ microspheres composed of 25 nm thick nanosheets (Figure 2d) were synthesized via a surfactant-assisted hydrothermal procedure in [76]. The cathode produced from V_2_O_5_ microspheres with an average diameter of 3 μm delivered the initial discharge capacity of 126.2 mAh·g^−1^ at 50 mA·g^−1^ in 0.25 M Mg(AlCl_2_EtBu)_2_/THF, demonstrated good cycling stability (90.7 mAh g^−1^ after 80 cycles) and enhanced rate capability (60 mAh·g^−1^ at 200 mA·g^−1^). The improved electrochemical performance of the V_2_O_5_ microflowers was explained by the increased specific surface area (13.7 m^2^·g^−1^) and flexibility. The microspheres with larger size (7 and 15 μm) or structural irregularities demonstrated lower capacities and cycling stability (Figure 2e). The charge–discharge mechanism was investigated by X-ray diffraction and X-ray photoelectron spectroscopy.

Monodisperse spherical V_2_O_5_ particles with a diameter of 230–250 nm were obtained in [77] (Figure 2f). The V_2_O_5_ spheres exhibited an initial discharge capacity of 225 mAh·g^−1^ (Figure 2g) and a stabile discharge capacity of 190 mAh·g^−1^ at 10 mA·g^−1^ in a dry Mg(ClO_4_)_2_/AN electrolyte. The rate performance of V_2_O_5_ spheres (Figure 2h) and long-term cycling stability at different current rates were good, and the specific discharge capacity of 55 mAh·g^−1^ was achieved at 50 mA·g^−1^ with 5% and 13% capacity fading after 50 and 100 cycles, respectively. The retention of 95% coulombic efficiency after 100 cycles and the stability of the phase and morphology confirmed the stability of the material during Mg^2+^-ion intercalation/deintercalation.

**Figure 3:**
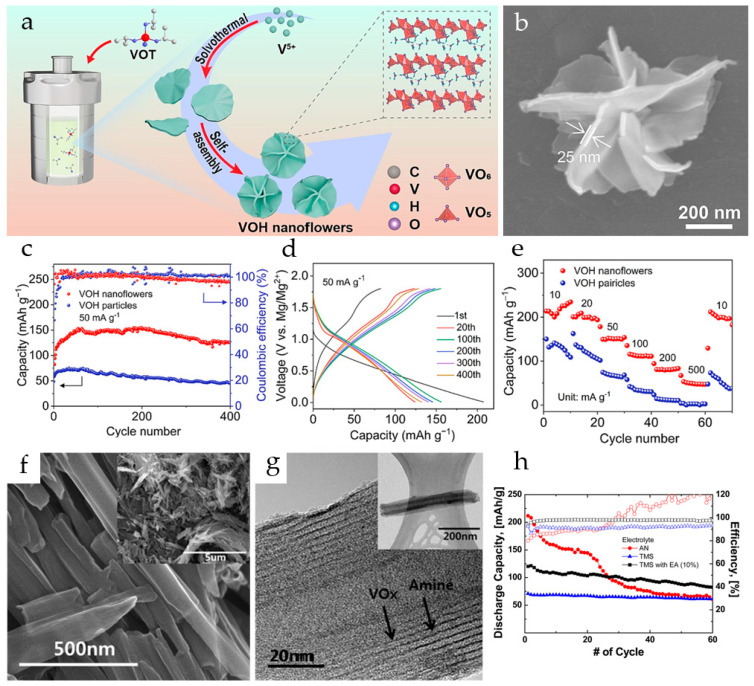
(**a**) Scheme of the preparation of V_5_O_12_·6H_2_O nanoflowers, (**b**) SEM of a V_5_O_12_·6H_2_O nanoflower, (**c**) cycling performance of V_5_O_12_·6H_2_O nanoflowers and nanoparticles at 50 mA·g^−1^, (**d**) discharge/charge profiles of V_5_O_12_·6H_2_O nanoflowers, (**e**) rate capability of V_5_O_12_·6H_2_O nanoflowers and nanoparticles. (Reprinted with permission from [78]. Copyright 2023, Elsevier). (**f**,**g**) High-resolution and (inset) low-resolution SEM and TEM images of VO_x_-NTs, (**h**) discharge capacity and coulombic efficiency of VO_x_-NT electrodes in AN, TMS, and TMS-EA electrolytes. (Reprinted with permission from [82]. Copyright 2016, American Chemical Society).

V_5_O_12_·6H_2_O nanoflowers formed by self-assembly of nanosheets were obtained by a one-step solvothermal method (Figure 3a) in [78]. The synthesized V_5_O_12_·6H_2_O had uniform morphology with an average size of a nanoflower of 650 nm while the individual nanosheets were about 25 nm thick (Figure 3b). For comparison, V_5_O_12_·6H_2_O nanoparticles were also synthesized. The galvanostatic tests revealed that both V_5_O_12_·6H_2_O nanoflowers and nanoparticles showed a similar activation process, the nanoparticles having a much lower capacity (Figure 3c). It was shown that the structural water in the interlayer of V_5_O_12_·6H_2_O improved the stability of the crystal structure and created more active sites for Mg^2+^ storage. The nanoflower morphology increased the active surface area, and enhanced the contact between the electrode material and the electrolyte, improving the diffusion kinetics of Mg^2+^. The V_5_O_12_·6H_2_O nanoflowers had a high initial specific capacity of 234.3 mAh·g^−1^ and reversible capacity after activation of 153.2 mAh·g^−1^ at 10 mA·g^−1^ (in a 1:1 mixture of 0.8M PhMgCl and 0.4M AlCl_3_ in anhydrous THF electrolyte) (Figure 3d), a good rate capability in the current range 50–500 mA·g^−1^ (Figure 3e), and long cycling stability (73.8% capacity retention after 1500 cycles at 100 mA·g^−1^, i.e., 0.017% capacity loss per cycle). It was proposed that the layered structure of the V_5_O_12_·6H_2_O nanoflowers can accommodate the volume changes during the intercalation/deintercalation of Mg^2+^, and the interlayer water molecules can serve as pillars enhancing the structural stability.

Ultrathin 2D V_6_O_13_ nanosheets were synthesized via a microwave-assisted method [79]. Two pairs of redox peaks observed in CVs at 0.9/0.68 V and 1.15/1.33 V (vs. Mg/Mg^2+^) correspond to the magnesiation/demagnesiation process, suggesting that it is a two-step process. The V_6_O_13_ nanosheet cathode delivered a maximum discharge specific capacity of 324 mAh·g^−1^ at 20 mA·g^−1^ in 1 M Mg(ClO_4_)_2_/AN, corresponding to 3.22 mol of Mg^2+^ ions’ insertion per unit formula of V_6_O_13_. At current densities of 40, 60, and 80 mA·g^−1^, the reversible specific capacities were 278, 244, and 214 mAh·g^−1^, respectively, indicating the good rate capability of the material. A high reversible capacity of 200 mAh·g^−1^ was retained after 30 cycles at 40 mA·g^−1^ with a ~100% coulombic efficiency.

Vanadium oxide nanotubes are advantageous for Mg^2+^-intercalation due to their large interlayer distance, open ends, and large inner and outer diameters. The shape of the tubes is favorable for an ion insertion process because the diffusion path in the solid is shorter and the heterogeneous kinetics are faster at higher surface-to-bulk ratios. In addition, the tubes can provide electrolyte-filled channels or voids for fast Mg^2+^-ion transportation to the insertion sites.

VO_x_ nanotubes (with the nominal composition of VO_2.37_[C_18_H_40_N]_0.26_) were obtained from V_2_O_5_ and octadecylamine through a hydrothermal reaction [80]. The nanotubes had open ends and were 1–3 μm long, and their outer/inner diameter was 60–100 nm/15–40 nm, respectively. In an 0.25 M Mg(AlBu_2_Cl_2_)_2_/THF electrolyte, the material exhibited good cycle performance, and the initial specific discharge capacity was 81 mAh·g^−1^ at 5 mA·g^−1^. The Mg^2+^-insertion into the VO_x_ nanotubes was confirmed by the XPS and XPD results.

VO_x_ nanotubes were obtained using a microwave-assisted hydrothermal method and low and high concentrations of the amine template, denoted as HT and LT, respectively [81]. The amount of the amine template did not affect the morphology of the VO_x_ nanotubes; both types of nanotube had an average outer diameter of 80–100 nm and were 1–5 μm long. The performance of VO_x_ nanotubes with various oxidation states (V^3+^/V^4+^/V^5+^) as a cathode for RMB was studied in 0.5 M Mg(ClO_4_)_2_/AN solution. The kinetics of magnesium insertion and extraction depended highly on the oxidation state and bonding structure of the nanotubes’ surface. The formation of V^3+^-ions in highly reduced VO_x_ nanotubes resulted in high initial discharge capacity (218 mAh·g^−1^ for HT-VO_x_ and 230 mAh·g^−1^ for LT-VO_x_ at 0.2 C (60 mA·g^−1^)), excellent cycling performance, and lower charge transfer resistance at the electrode/electrolyte interface compared to VO_x_ nanotubes containing vanadium ions of higher oxidation states. Although the initial capacities of HT-VO_x_ were lower than those of LT-VO_x_, HT-VO_x_ demonstrated better cycling performance, with capacity retention of 70.8% after 20 cycles. The coulombic efficiency of both materials increased while cycling.

The Mg^2+^-ion insertion/extraction performance of VO_x_-NTs was investigated in Mg(ClO_4_)_2_ tetramethylsilane-ethyl acetate (TMS-EA) electrolyte in comparison with an AN electrolyte [82]. The VO_x_-NTs were prepared using a microwave-assisted hydrothermal method starting with V_2_O_5_ and octadecylamine. As seen in the SEM and TEM images (Figure 3f,g), the VO_x_-NTs were open-ended, multilayered tubular structures, with alternating arrangements of VO_x_ and amine layers. VO_x_-NTs showed an initial capacity of more than 200 mAh·g^−1^ in de-aerated 0.5 M Mg(ClO_4_)_2_/AN electrolyte solution, but had poor capacity retention. Superior cycling performance and coulombic efficiency were observed for the VO_x_-NTs in TMS and TMS-EA, although the initial discharge capacities in TMS-based electrolytes were lower (Figure 3h). It was shown that the TMS-EA solvent improved the cell performance due to the higher stability of TMS against oxidation and the strong Mg^2+^ coordination ability of EA. The initial discharge capacity of VO_x_-NT in TMS-EA solution was 124 mAh·g^−1^ at 0.2 C (60 mA·g^−1^), and the capacity retention was 70% after 80 cycles.

The electrochemical Mg^2+^-ion intercalation and extraction in multi-walled vanadium oxide nanotubes VO_x_-NTs was investigated in [83]. The VO_x_-NTs had an open-ended structure of multilayer scrolls with an outer diameter of 50 nm and a length of a few µm, and the VO_x_ interlayer spacing was 2.7 nm. The initial specific discharge capacity for VO_x_-NTs in 1 M Mg(ClO_4_)_2_/AN and in 1 M Mg(TFSI)_2_/G2 was 146 ± 36 and 33 ± 12 mAh·g^−1^ at 5 mA·g^−1^, respectively, since, as a rule, the cells with the Mg(TFSI)_2_/G2 electrolyte provide lower initial capacities compared to Mg(ClO_4_)_2_/AN. In both electrolytes, the VO_x_-NTs demonstrated poor capacity retention. The intercalation of Mg^2+^-ions was confirmed by ICP-AES. *In operando* scattering studies have shown that Mg^2+^-ion intercalation into VOx-NTs is accompanied by an increase in the interlayer spacing and a loss of the long-range stacking order.

### 2.3. Bilayer V_2_O_5_

V_2_O_5_ gels can be synthesized by a sol–gel process from a variety of precursors. Xerogels and aerogels differ in the drying process that removes the solvent from the nanoscale pores and capillaries after the material is synthesized. Xerogels are dried at ambient conditions, resulting in a more compact and dense material than aerogels. Aerogels are dried by filling the pores and capillaries with a supercritical fluid and then cooling so that the pores are filled with a gas. This process allows the pores to remain intact and increases the surface area of the material. V_2_O_5_ aerogel has a surface area ~30 times larger than that of V_2_O_5_ xerogel [27].

V_2_O_5_ gels have an enhanced capacity for insertion/extraction of magnesium ions due to their water content. Furthermore, the increased surface area supports more efficient diffusion and provides more sites for insertion/extraction of magnesium ions, thus increasing the ability of the material to enable high-rate charge and discharge. In addition, the capacity increases due to the large surface area because there is less isolated bulk material that the intercalating ions cannot reach.

Amorphous V_2_O_5_ aerogels with high surface area, consisting of interpenetrating networks of water and V_2_O_5_ ribbons, are capable of accommodating 0.6–2 moles of Mg^2+^ per mole of V_2_O_5_ as a result of chemical insertion [84], or two moles formally corresponding to the reduction of V^5+^ to the V^3+^ state [85].

Bilayer V_2_O_5_·*n*H_2_O is generally considered to be amorphous due to the lack of long-range structural order, but at the nanoscale, a well-organized repetition of bilayers is observed. The synthesis route affects the morphology of V_2_O_5_·*n*H_2_O. The reduced structural order, wide interlayer space, and short diffusion length in V_2_O_5_·*n*H_2_O allow reversible cation accommodation [86]. The interlayer structural water can increase the interlayer spacing, which is favorable for the intercalation of Mg^2+^. The structural water molecules can partially shield the charge of Mg^2+^, which leads to the improvement in the diffusion kinetics of Mg^2+^ in bilayer V_2_O_5_ [86].

The insertion of Mg^2+^ into V_2_O_5_ xerogel and V_2_O_5_ xerogel/C composite in aqueous Mg(NO_3_)_2_ electrolytes was studied [87]. The intercalation/deintercalation reaction of Mg^2+^- ions was much faster in the case of the V_2_O_5_ xerogel/C composite as compared to the pure V_2_O_5_ xerogel. The addition of 10% carbon black during the synthesis of V_2_O_5_ xerogel/C composite allowed a significant improvement in the electrochemical behavior of this electrode material; the specific capacity of V_2_O_5_ xerogel/C composite in Mg(NO_3_)_2_ was 107 mAh·g^−1^, while pure V_2_O_5_ xerogel yielded 50 mAh·g^−1^.

V_2_O_5_ xerogel was prepared under microwave (MW) irradiation [88]. The structure and electrochemical properties of the V_2_O_5_ xerogel were compared with those of V_2_O_5_ prepared by conventional heat treatment. XRD revealed that the V_2_O_5_ xerogel prepared by MW irradiation was low-crystalline. The first discharge capacity of V_2_O_5_ prepared by MW irradiation was 175 mAh·g^−1^ at a 0.1 C rate in 0.3 M Mg(ClO_4_)_2_/PC solution, and the second discharge capacity increased to 463 mAh·g^−1^, indicating that Mg^2+^-insertion increased after the first cycle. The capacity of V_2_O_5_ prepared by heat treatment at 200 °C was 138 and 190 mAh·g^−1^ for the first and the second cycles, respectively, and the capacity of V_2_O_5_ prepared by heat treatment at 300 °C was 77 mAh·g^−1^.

An amorphous V_2_O_5_ xerogel/graphite composite containing 10 wt.% synthetic graphite showed an initial deintercalation capacity of 77 mAh·g^−1^ at 10 mV·s^−1^ (~18 C rate) in Mg(NO_3_)_2_ aqueous electrolyte [89]. After ten cycles, the value decreased to 63.5 mAh·g^−1^. In GCD tests, a relatively high magnesium storage capacity of 62, 53, 47, and 44 mAh·g^−1^ was obtained at 2, 3, 4, and 5 A·g^−1^, respectively.

**Figure 4:**
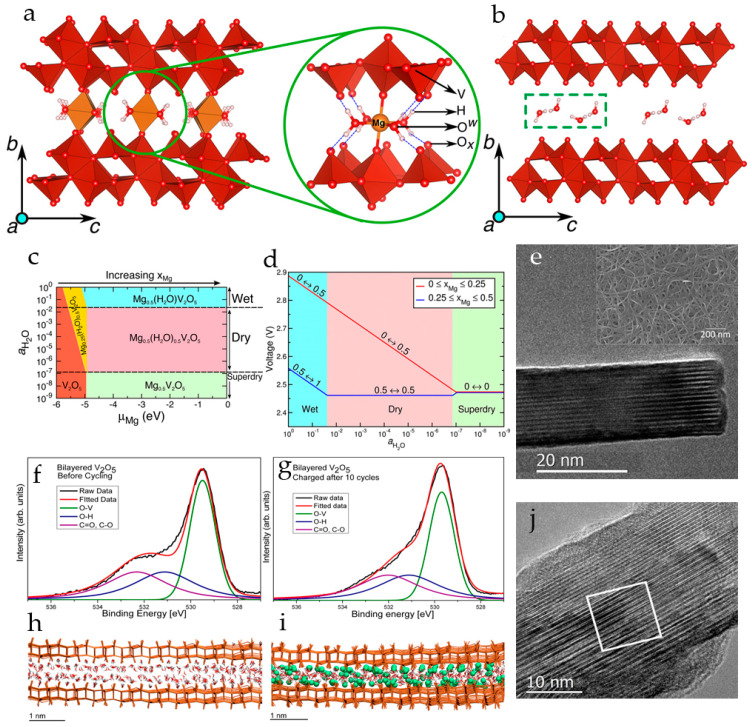
Structures of (**a**) fully magnesiated (xMg = 0.5) and (**b**) fully demagnesiated xerogel with one H_2_O molecule per formula unit of V_2_O_5_, (**c**) potential phase diagram at 0 K of Mg-intercalated V_2_O_5_ xerogel as a function of water content in the electrolyte and Mg chemical potential (μMg = 0 corresponds to full magnesiation), (**d**) average Mg insertion voltage for low and high Mg concentrations as a function of water content in the electrolyte (*a*H_2_O). (Reprinted with permission from [90]. Copyright 2016, American Chemical Society). (**e**) HRTEM of a nanoribbon of bilayered V_2_O_5_ (inset—SEM of the nanoribbon architecture on a carbon nanofoam substrate), (**f**,**g**) XPS spectra of bilayered V_2_O_5_ before and after 10 cycles of charging with Mg^2+^-ions, and (**h**) MD simulation of the V_2_O_5_ bilayer immersed in water and (**i**) V_2_O_5_ bilayer (V in the +4 state) in the presence of Mg^2+^ ions in the presence of water, (**j**) HRTEM image of Mg-enriched V_2_O_5_. (Reprinted with permission from [91]. Copyright 2015, American Chemical Society).

The scenario of Mg^2+^ and H_2_O co-intercalation in nanocrystalline V_2_O_5_ xerogel was analyzed by first-principles calculations [90]. The models of fully relaxed structures of the fully magnesiated and demagnesiated V_2_O_5_ xerogel, where two individual V_2_O_5_ layers are bound by long interlayer V–O bonds and the intercalated Mg atoms and H_2_O molecules are located in the space between two bilayers, are shown in Figure 4a,b.

Analysis of the stable phases of Mg-intercalated V_2_O_5_ xerogel at different voltages and in electrolytes with different water contents revealed a range of concentrations of intercalated Mg-ions in the V_2_O_5_ xerogel and H_2_O in the electrolyte where there is no thermodynamic driving force for the water molecules to shuttle with Mg^2+^-ions during electrochemical cycling (Figure 4c,d). It was also demonstrated that shuttling of water molecules with the Mg^2+^-ions in wet electrolytes yields higher voltages than in dry electrolytes.

Highly hydrated nanoribbons of bilayer V_2_O_5_ with a large interlayer distance (13.5 Å) were electrodeposited on porous highly conductive carbon nanofoam [91]. This method also allowed effective incorporation of defects, water, and hydroxyl groups, which in turn promoted electron transfer and (de)intercalation of highly charged Mg^2+^ ions. The “as-prepared” individual V_2_O_5_ nanoribbon is shown in an HRTEM image in Figure 4e. Mg^2+^ ions were incorporated into the as-prepared bilayer V_2_O_5_ cathodes by galvanostatic discharge at 20 μA to the potential 0.2 V (vs. Mg/Mg^2+^). The specific capacity of 240 mAh·g^−1^ obtained after this preconditioning procedure suggests reduction of vanadium V^5+^ to V^4+^. The electrode demonstrated reversible Mg^2+^ (de)intercalation in 1 M Mg(ClO_4_)_2_/AN electrolyte with a capacity of 150 mAh·g^−1^ at C/15 rate. The O 1s XPS spectra before cycling and after 10 cycles of charging showed that strongly bound structural hydroxyl groups remain in the structure of bilayer V_2_O_5_ during cycling (Figure 4f,g), and act as a lubricant for reversible (de)intercalation of solvated Mg^2+^ ions between the layers of V_2_O_5_. Molecular dynamics simulations of a three-bilayer V_2_O_5_ model, shown in Figure 4h,i, suggested that upon insertion of Mg^2+^-ions into the V_2_O_5_ structures, the spacing between bilayers decreases from ~12 to ~11 Å due to the interaction of Mg^2+^-ions with bilayer apical oxygen and structural hydroxyl groups, but a significant amount of H_2_O molecules remains in the structure, solvating the Mg^2+^ ions. The HRTEM image (Figure 4j) showed that after Mg^2+^-ions were intercalated, the layered V_2_O_5_ structure featured a spacing of 6.0 to 6.4 Å, and the bilayer distance was not uniform across the whole imaged area.

The reversible insertion/extraction of Mg^2+^ into the V_2_O_5_·*n*H_2_O electrode accompanied by the co-intercalation of solvent molecules was demonstrated [92]. The V_2_O_5_·*n*H_2_O achieved a discharge capacity of 50 mAh·g^−1^ (0.25 Mg per V_2_O_5_·*n*H_2_O) in a Mg(TFSI)_2_/G2 (diglyme) electrolyte at a current density of 20 mA·cm^−2^. It was shown that the process of Mg^2+^ intercalation into V_2_O_5_·*n*H_2_O involves the formation of new phases, while the bilayer spacing expands/contracts. The large interlayer spacing of bilayer V_2_O_5_ allowed the reversible co-intercalation of Mg^2+^ and solvent molecules.

Bilayered nanostructured V_2_O_5_·*n*H_2_O 2D nanopapers with oxygen defects (BL-HVOd NPS) with improved Mg^2+^ insertion/extraction kinetics were obtained [93]. The interlayer water molecules effectively stabilized the expanded interlayer spacing (10.6 Å) in BL-HVOd NPS and shielded the electrostatic interaction between Mg^2+^-ions and BL-HVOd NPS lattice, improving diffusion kinetics during repeated cycling (the log(*D*_Mg_^2+^) value was in the range from −8.3 to −9.5 during charge and discharge). The nanopaper structure of BL-HVOd NPS enhanced electrolyte/electrode contact and reduced the diffusion path of Mg^2+^-ions, improving the rate performance. Cells with a BL-HVOd NPS cathode, AC anode, and 2 M Mg(CF_3_SO_3_)_2_ aqueous electrolyte demonstrated a reversible capacity of 162.8 mAh·g^−1^ at 0.2 A·g^−1^ and high cyclic stability with 88% capacity retention after 2000 cycles at 10 A·g^−1^. This was attributed to the contribution of oxygen defects.

Bilayer V_2_O_5_ is a promising cathode material for RMBs, but the low electronic conductivity of bilayer V_2_O_5_ is still a challenge that hinders its further application in RMBs. The main strategy to solve this problem is the modification of V_2_O_5_ with highly conductive materials or the pre-intercalation of metal ions to improve the conductivity of bilayer V_2_O_5_ and promote the ionic and electronic transport.

### 2.4. Nanocomposites of V_2_O_5_ with Conductive Carbons

The use of conductive carbons as additives for the improvement in electrical conductivity of V_2_O_5_ has also been studied in a large number of works.

Mg^2+^ ion intercalation into a V_2_O_5_/carbon composite, consisting of aggregated carbon particles (30–100 nm) covered with a thin layer (<100 nm) of V_2_O_5_ xerogel, in 1 M Mg(ClO_4_)_2_/AN electrolyte, was reported in [94,95]. Mg^2+^ insertion into V_2_O_5_ xerogel/C composites and accompanying structural changes were studied [94]. In the cyclic voltammogram for the V_2_O_5_ xerogel/C composite in 1 M Mg(ClO_4_)_2_/AN, two broad cathodic peaks associated with the Mg^2+^-insertion process were observed, pointing at the existence of two different Mg intercalation sites in the V_2_O_5_ xerogel. *Ex situ* FT-IR spectra suggested that Mg^2+^ is inserted into inner layer sites of V_2_O_5_ at −0.15 V and into the interlayer sites at −0.65 V vs. Ag/Ag^+^. The reversibility of Mg^2+^ intercalation/deintercalation was confirmed by FT-IR and XRD. The V_2_O_5_/carbon composite showed higher capacity than a conventional V_2_O_5_ electrode. It was assumed that the Mg^2+^ diffusion into the interlayer is slow, but since the V_2_O_5_/C composite has a large interlayer distance and short diffusion length compared to the normal xerogel of V_2_O_5_, the interlayer site could be utilized more effectively for Mg^2+^-insertion.

With a V_2_O_5_ gel/carbon composite, insertion of 1.84 mol Mg per 1 mol V_2_O_5_ in the first cycle at a scan rate of 0.1 mV·s^−1^ was found, resulting in a specific capacity of 540 mAh·g^−1^ (per V_2_O_5_ mass) [95]. In GCD tests, at a current density of 1.0 A·g^−1^ the composite electrode showed a capacity of about 600 mAh·g^−1^, and at a high current density of 20 A·g^−1^ the capacity of about 300 mAh·g^−1^ was maintained. The capacity of the carbon-free V_2_O_5_ xerogel electrode at 1.0 A·g^−1^ was only 150 mAh·g^−1^, and at 20 A·g^−1^ it was less than 10% of the capacity of the composite electrode. Thus, homogeneous mixing of a V_2_O_5_ sol with carbon particles, resulting in the formation of a thin layer of V_2_O_5_ gel around the carbon particles, was effective in improving the Mg^2+^ intercalation behavior. The reduction in the Mg^2+^ diffusion length in V_2_O_5_ and the improvement in the electronic conductivity by the carbon particles resulted in high capacities, especially at high current densities. The composites of vanadium oxide with graphene are expected to advance the RMB performance by providing rapid electron transport and easy Mg^2+^-diffusion due to the large surface area and excellent structural stability.

The synthesis of V_2_O_5_/graphene (GNP) nanoparticles by a ball milling technique was attempted in [96] to enhance the electrochemical performance of V_2_O_5_. The full cell with a Mg anode, (V_2_O_5_)_1-x_/GNP_x_ cathode, and MgNO_3_∙6H_2_O/tetraethylene glycol dimethyl ether electrolyte yielded an initial capacity of ~90 mAh·g^−1^ at a low current density, while Mg/V_2_O_5_ cells exhibited the initial discharge capacity of ~100 mAh·g^−1^ under the same testing conditions. The capacities dropped to half the initial value on the second cycle and then the cells short-circuited, probably due to the incompatible electrolyte.

A composite of V_2_O_5_ with graphene oxide (GO/V_2_O_5_) was prepared by a solvothermal method [97]. SEM revealed that the V_2_O_5_ microparticles were wrapped around GO sheets, and TEM micrographs demonstrated the tight contact between GO and V_2_O_5_ microparticles. The good contact and dispersion of the composite components provided improved electrochemical performance of the material. The GO/V_2_O_5_ cathode was tested in coin cells with a Mg foil anode and 0.25 M Mg(AlCl_2_EtBu)_2_/THF as an electrolyte. The GCD measurements showed an initial discharge capacity of GO/V_2_O_5_ of 178 mAh·g^−1^ at 0.2 C, which faded to 160 and 150 mAh·g^−1^ in the second and third cycle, respectively, due to formation of a Mg passivation layer, but still remained at 140 mAh·g^−1^ after 20 cycles.

**Figure 5:**
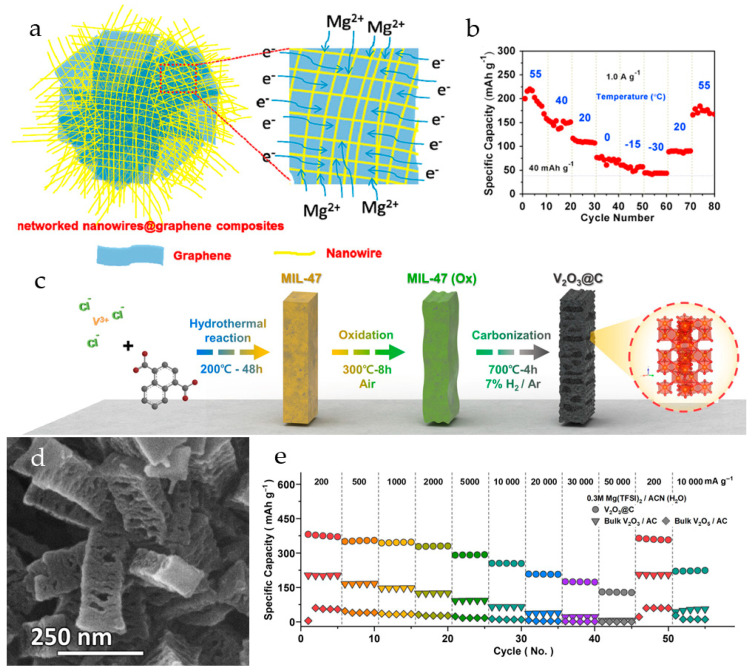
(**a**) Scheme of V_2_O_5_·*n*H_2_O@rGO nanocomposite with electron/ion transport pathways, (**b**) cycling performance of V_2_O_5_·*n*H_2_O@rGO at 1.0 A·g^−1^, (**c**) discharge capacity V_2_O_5_·*n*H_2_O@rGO at various temperatures at 1.0 A·g^−1^. (Reprinted with permission from [98]. Copyright 2015, Elsevier). (**c**) Scheme of the synthesis of V_2_O_3_@C from MOFs, (**d**) SEM image of V_2_O_3_@C, (**e**) rate capabilities of V_2_O_3_@C, bulk-V_2_O_3_/AC and bulk-V_2_O_5_/AC. (Reprinted with permission from [100]. Copyright 2024, John Wiley and Sons).

rGO-decorated hydrated V_2_O_5_ nanowire composites with different contents of crystal water have been synthesized by a sol–gel method with subsequent freeze-drying [98]. The V_2_O_5_·nH_2_O/rGO aerogel with a water content of 12.3% and large lattice spacing of 1.13 nm had a highly porous, interconnected 3D structure with V_2_O_5_ nanowires anchored on rGO (Figure 5a). The V_2_O_5_·*n*H_2_O/rGO electrode showed a capacity of 280 mAh·g^−1^ at 100 mA·g^−1^ in a 0.5 M Mg(TFSI)_2_/AN electrolyte, a capacity retention of 81% after 200 cycles, and a coulombic efficiency of 99%. At 50 mA·g^−1^ the capacity was 320 mAh·g^−1^. The nanocomposite displayed a wide working temperature range (~ 30–55 °C). As shown in Figure 5b, at 55 °C a high discharge capacity of ~200 mAh·g^−1^ was achieved, much higher than the capacity of 120 mAh·g^−1^ at room temperature. A reversible capacity of 40 mAh·g^−1^ was delivered at −30 °C. The improved electrochemical performance of the material was attributed to the shielding effect of crystal water as well as the synergistic effects of rGO, which provided Mg diffusion pathways, high surface area, and structural stability.

A three-dimensional structure of V_2_O_3_ nanoparticles with reduced graphene oxide (rGO) with improved conductivity and structural stability was prepared by spray-drying of a dispersed solution [99]. In the 0.3 M Mg(TFSI)_2_/AN electrolyte solution, V_2_O_3_@rGO microspheres demonstrated a high specific capacity (291.3 mAh·g^−1^ at 50 mA·g^−1^) and improved rate performance (185.3 mAh·g^−1^ at a high current density of 2 A·g^−1^) compared to pure V_2_O_3_ (~80 mAh·g^−1^). Excellent cycling stability (88.5% capacity retention after 1000 cycles at 0.5 A·g^−1^) with coulombic efficiency close to 100% was achieved. V_2_O_3_@rGO delivered a capacity of 472.1 mAh·g^−1^ in the GITT study, and the calculated diffusion coefficient of Mg^2+^ in the V_2_O_3_@rGO structure was 3.4 × 10^−11^ cm^2^·s^−1^.

A hierarchical V_2_O_3_@C structure with an accordion-like vanadium oxide/carbon heterointerface was synthesized from vanadium-based metal organic frameworks (Figure 5c) [100]. The mesoporous V_2_O_3_@C nanorods had a length of 250–300 nm, elongated pores of 30–50 nm (Figure 5d), and a BET surface area of 114.6 m^2^·g^−1^. The V_2_O_3_@C demonstrated a capacity of 354.8 mAh·g^−1^ at a current density of 500 mA·g^−1^ and an ultra-high capacity of 130.4 mAh·g^−1^ at 50 A·g^−1^ in 0.3 M Mg(TFSI)_2_/AN(H_2_O) with a coulombic efficiency of 99.6% and a capacity retention of 60.0% (1000 cycles, 500 mA·g^−1^). To verify the advantages of hierarchical accordion-like heterointerfaces, the rate performance of the V_2_O_3_@C electrodes was compared with that of bulk-V_2_O_3_/AC and bulk-V_2_O_5_/AC electrodes prepared with activated carbon in the same ratio as in V_2_O_3_@C. As shown in Figure 5e, the specific capacity of bulk-V_2_O_3_/AC at 200 mA·g^−1^ (203.2 mAh·g^−1^) is four times higher than of bulk-V_2_O_5_/AC (54.9 mAh·g^−1^) due to the anodic hydration of the V_2_O_3_ phase, occurring in bulk-V_2_O_3_/AC upon the first charging. The crystalline structure of V_2_O_3_ was reconstructed into a V_3_O_7_∙H_2_O@C through an anodic hydration reaction upon the first cycle. The cell was disassembled under an inert atmosphere and the V_3_O_7_∙H_2_O@C electrode was retested in a 0.3 M Mg(TFSI)_2_/AN electrolyte, where the specific capacity of the V_3_O_7_∙H_2_O@C electrode was 259.3 mAh·g^−1^ at 500 mA·g^−1^. The capacity difference of 33.9 mAh·g^−1^ between the two electrolytes indicated that the capacity contributed by protons in the water-containing electrolyte is about 13% of the total capacity. The higher overpotential of the V_3_O_7_∙H_2_O@C electrode in water-free electrolyte solution resulted from the slower diffusion of Mg^2+^-ions. In a full cell with a Mg anode and a 0.3 M Mg(TFSI)_2_  +  0.25 M MgCl_2_/DME electrolyte within the voltage range of 2.25 V vs. Mg/Mg^2+^, the V_2_O_3_@C exhibited a capacity of 245.1 mAh·g^−1^.

### 2.5. Foreign Cation Pre-Intercalated Vanadium Oxides

The rich valence state variation of vanadium and the easy distortion of V–O polyhedra lead to the ability of V–O structure adaptation to incorporate different cations. The resulting derivatives with an A–V–O structures (A—metal ion or ${\mathrm{NH}}_{4}^{+}$) are called vanadates or foreign cation pre-intercalated vanadium oxides.

The pre-intercalation strategy has been widely recognized as a way to improve the properties of vanadium oxide cathodes for RMBs by increasing the interlayer space due to the pillaring effect combined often with improved stability of the obtained structures, allowing reversible insertion/extraction of Mg^2+^-ions. The analysis of the results of different works shows that, in most cases of pre-intercalated ions, a dual effect of structure expansion and void hydration takes place.

It should be noted that the pre-intercalation of cations, with its positive effects on increasing the interlayer spacing of V_2_O_5_ bilayers, also has some drawbacks. The most important one is that the introduction of electrochemically inactive cations leads to a decrease in specific capacitance. Therefore, the necessary optimization of cathode materials requires a balance between structural and stability factors on the one hand, and the improvement of specific capacities on the other hand.

The differences in the active material composition (especially water content), electrode processing and morphology, and electrolyte system make it difficult to compare results from different groups.

Among the cations used for pre-intercalation of vanadium oxides for RMBs are mono-, bi-, and tri-valent ions: Na^+^ [101,102,103,104], ${\mathrm{NH}}_{4}^{+}$ [105,106], Mg^2+^ [107,108,109,110], Mn^2+^ [111], Ca^2+^ [112], and Al^3+^ [113].

**Figure 6:**
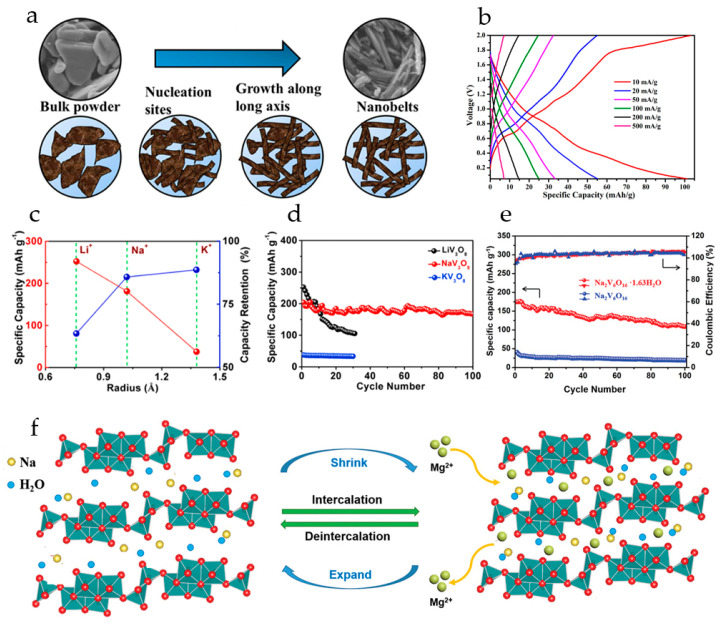
(**a**) Scheme of the growth mechanism of the NaV_3_O_8_·1.69H_2_O nanobelts from V_2_O_5_, (**b**) charge–discharge curves of NaV_3_O_8_·1.69H_2_O at current densities 10–500 mA·g^−1^. (Reprinted with permission from [102]. Copyright 2018 American Chemical Society). (**c**) Specific capacity of alkali ion (Li^+^, Na^+^, K^+^) pre-intercalated V_3_O_8_ at 100 mA·g^−1^ and the capacity retention after 30 cycles, (**d**) cycling performance of alkali ion pre-intercalated V_3_O_8_ at 100 mA·g^−1^. (Reprinted with permission from [101]. Copyright 2019, Elsevier). (**e**) Cyclic performance of Na_2_V_6_O_16_ and Na_2_V_6_O_16_·1.63H_2_O at 50 mA·g^−1^, (**f**) scheme of Mg^2+^ intercalation/deintercalation into Na_2_V_6_O_16_·1.63H_2_O during the charge–discharge processes. (Reprinted with permission from [104]. Copyright 2020, John Wiley and Sons).

NaV_3_O_8_·1.69H_2_O nanobelts were synthesized from commercial V_2_O_5_ powder by a solvothermal procedure under ambient conditions (Figure 6a) [102]. The cells with a Mg anode and all-phenyl complex electrolyte demonstrated an initial discharge capacity of 150 mAh·g^−1^ and 110 mAh·g^−1^ after five cycles at a current density of 10 mA·g^−1^. The GCD curves of NaV_3_O_8_·1.69H_2_O at different current densities (Figure 6b) showed several plateaus during the charging and discharge process, due to the multistep redox reactions responsible for the insertion/extraction of Mg^2+^ ions. The limited specific capacity of the cells was attributed to the trapping of Mg^2+^ ions in the lattice of NaV_3_O_8_. The cathodes demonstrated high cyclic stability with 80% capacity retention after 100 cycles at 50 mA·g^−1^.

Pre-intercalating of alkali ions (Li^+^, Na^+^, K^+^) into layered vanadium oxide was studied in [101]. It was shown that the interlayer spacing of Li-, Na-, K-intercalated V_3_O_8_ increased regularly with the size of the pre-intercalated cations. The cycling performance of cathodes for Mg^2+^ storage was enhanced with the bigger radius of pre-intercalated ion (Figure 6c); after 30 cycles the capacity retention of KV_3_O_8_ was 88.6%, compared to 85.78% of NaV_3_O_8_ and 42.2% of LiV_3_O_8_, although the specific capacity of KV_3_O_8_ was the lowest (37.56 mAh·g^−1^). The initial specific capacity of LiV_3_O_8_ and NaV_3_O_8_ at 100 mA·g^−1^ was 252.2 and 204.16  mAh·g^−1^, respectively (Figure 6d). Based on structural analysis and electrochemical tests, it was concluded that pre-intercalation with Na^+^ resulted in a more stable interlayer structure, allowing free Mg^2+^ diffusion and preventing destructive collapse of the layers during the charge–discharge.

The interlayer spacing of V_2_O_5_ was expanded in [103] by introducing Na^+^-ions in the crystal lattice. NaV_6_O_15_ (NVO) free of crystal water was synthesized and studied as a cathode material for RMBs with anhydrous 0.5 M Mg(ClO_4_)_2_/AN electrolyte solution. It was shown that the introduction of Na^+^-ions enhanced the diffusion kinetics of Mg^2+^ ions (*D*_Mg_^2+^ during the discharge process was in the range of 7.55 × 10^−13^ to 2.41 × 10^−11^ cm^2^·s^−1^ according to GITT), and improved the stability of the layered structure. NVO exhibited high initial discharge capacity of 213.4 mAh·g^−1^ at the current density of 10 mA·g^−1^, good capacity retention (87% after 100 cycles at 20 mA·g^−1^), and good rate capability. The *ex situ* XRD showed that the mechanism of Mg^2+^ storage in NVO is reversible intercalation/de-intercalation. The DFT calculation results indicated that during the intercalation process, Mg^2+^-ions tend to occupy the semi-occupied sites of Na^+^ in the NVO.

Water-pillared Na_2_V_6_O_16_·1.63H_2_O nanowires obtained in [104] displayed high performance in magnesium storage. In a cell with a Mg(TFSI)_2_/DME electrolyte and an AC anode, Na_2_V_6_O_16_·1.63H_2_O exhibited a high specific capacity of 175 mAh·g^−1^ at 0.05 A·g^−1^, long-term cycling performance, and ≈100% coulombic efficiency. Under the same testing conditions, annealed Na_2_V_6_O_16_ delivered a low specific capacity of only 40 mAh·g^−1^ (Figure 6e). Na_2_V_6_O_16_·1.63H_2_O cathodes demonstrated a discharge voltage plateau at around 2.0 V (vs. Mg/Mg^2+^), ascribed to the Mg^2+^ ion intercalation into the bilayer structure. According to *ex situ* XRD analysis, Na_2_V_6_O_16_·1.63H_2_O possesses a very stable structure for reversible Mg^2+^ ion intercalation/deintercalation. During the discharge, Mg^2+^-intercalation is accompanied by shrinkage of the interlayer space (Figure 6f). During subsequent charge–discharge processes, the Mg^2+^-ions can reversibly intercalate/deintercalate into the Na_2_V_6_O_16_·1.63H_2_O layers. The water molecules acting as “pillars” stabilize the layered structure and effectively shield the high charge density of Mg^2+^-ions.

Magnesiated V_2_O_5_ xerogel with the formula Mg_0.1_V_2_O_5_·1.8H_2_O was synthesized in [109] by a low-temperature procedure. The material had an initial discharge capacity of 300 mAh·g^−1^ in 0.5 M Mg(TFSI)_2_/AN. A capacity of ~250 mAh·g^−1^ was maintained over eight cycles at a C/10 rate, consistent with an insertion of 1 equivalent of Mg^2+^ per formula unit. No change in the interlayer distance (12.3 Å) was observed upon cycling of Mg_0.1_V_2_O_5_·1.8H_2_O electrodes.

Mg-inserted V_2_O_5_·xerogel was prepared via an ion removal sol–gel method in [114]. The Mg_0.1_V_2_O_5_·2.35H_2_O cathode displayed a high electrode potential of 3.0 V (vs. Mg/Mg^2+^), high energy density of 420 mWh·g^−1^, and a discharge capacity of 140 mAh·g^−1^ at a 0.1 C rate.

**Figure 7:**
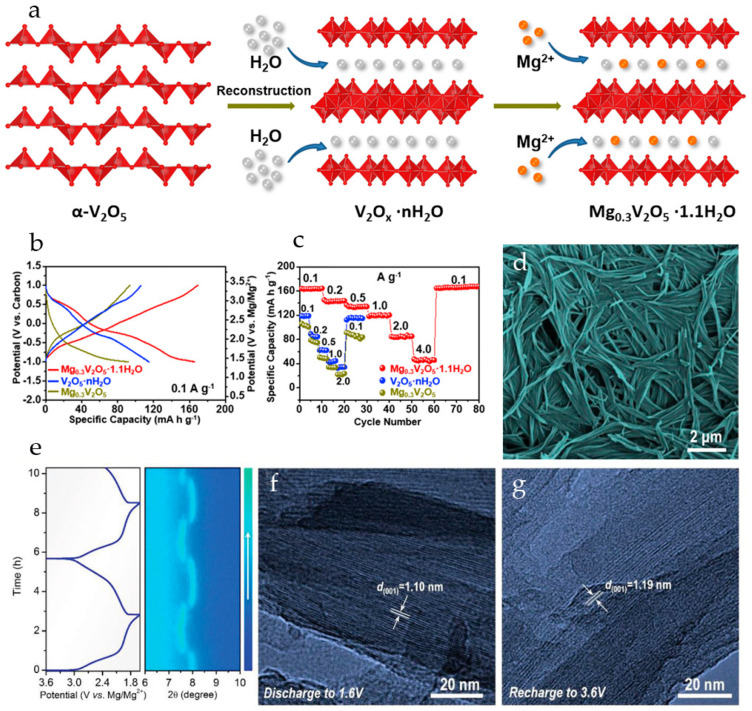
(**a**) Scheme of consecutive incorporation of water and magnesium to form the bilayer Mg_0.3_V_2_O_5_·1.1H_2_O, (**b**) charge–discharge curves, and (**c**) rate performance of Mg_0.3_V_2_O_5_·1.1H_2_O, V_2_O_5_·nH_2_O, and Mg_0.3_V_2_O_5_ (Reprinted with permission from [110]. Copyright 2019, Elsevier). (**d**) SEM image of MgVOH electrode, (**e**) *in situ* XRD contour map of the Mg_x_V_5_O_12_·*n*H_2_O electrode and corresponding high-resolution TEM images of (**f**) discharged and (**g**) recharged Mg_x_V_5_O_12_·*n*H_2_O electrode (Reprinted with permission from [108]. Copyright 2020, John Wiley and Sons).

Mg^2+^ pre-intercalated hydrated vanadium oxide nanowires with bilayer structure, Mg_0.3_V_2_O_5_·1.1H_2_O, were synthesized in [110] from α-V_2_O_5_ using a hydrothermal approach. The reaction of α-V_2_O_5_ with H_2_O_2_ lowers the valence state of V, resulting in structural transformation of VO_5_ square pyramids to VO_6_ octahedra, and a large number of H_2_O molecules are embedded into the interlayer. This results in a bilayer structure with a larger interlayer space, providing ample channels for the subsequent insertion of Mg^2+^ ions. As a result of the coordination between Mg^2+^ and lattice oxygen, the insertion of Mg^2+^ ions into the layers leads to layer slippage and shrinkage (Figure 7a). In order to investigate the role of Mg^2+^ ions and lattice water molecules, V_2_O_5_·nH_2_O and Mg_0.3_V_2_O_5_ nanowires were also prepared. In Mg_0.3_V_2_O_5_·1.1H_2_O, the intercalated Mg^2+^ ions work together with crystal water to provide wide channels for electrolyte ion transport during the charge–discharge process. Mg_0.3_V_2_O_5_·1.1H_2_O possessed high electronic conductivity, fast Mg^2+^ reaction kinetics, and good structural stability. The charge and discharge curves of Mg_0.3_V_2_O_5_·1.1H_2_O, V_2_O_5_·*n*H_2_O and Mg_0.3_V_2_O_5_ at 0.1 A·g^−1^ in a cell with 0.3 M Mg(TFSI)_2_/AN electrolyte and an activated carbon anode are shown in Figure 7b. Mg_0.3_V_2_O_5_·1.1H_2_O exhibited three discharge voltage plateaus at 3.02, 2.25, and 1.40 V (vs. Mg/Mg^2+^), which can be attributed to the multi-step Mg^2+^ intercalation in the bilayer structure. Mg_0.3_V_2_O_5_·1.1H_2_O delivered high capacity (164 mAh·g^−1^ at 0.1 A·g^−1^), corresponding to the insertion of 0.5 Mg^2+^ ions per formula unit. V_2_O_5_·*n*H_2_O had a similar charge–discharge curve, with a lower discharge capacity of 114 mAh·g^−1^. For Mg_0.3_V_2_O_5_, no obvious plateaus were observed on the charge–discharge curve and it had the lowest discharge capacity (91 mAh·g^−1^). As can be seen in Figure 7c, Mg_0.3_V_2_O_5_·1.1H_2_O demonstrated good rate performance (50 mAh·g^−1^ at 4.0 A·g^−1^) and high cycling stability (capacity retention of 80.0% after 10,000 cycles at 2.0 A·g^−1^). In a full RMB cell with a Na_2_Ti_3_O_7_ anode and the Mg_0.3_V_2_O_5_·1.1H_2_O cathode, the specific capacity of about 62 mAh·g^−1^ at the current density of 100 mA·g^−1^ was achieved, and the battery demonstrated a stable voltage plateau of 1.5 V and a cycle life of 100 cycles.

Mg^2+^-pillared hydrated vanadium oxide Mg_x_V_5_O_12_·*n*H_2_O nanofibers (Figure 7d), with expanded interlayer spacing were obtained in [108]. Mg^2+^ pillars and structural H_2_O molecules stabilized the material framework and promoted Mg^2+^ diffusion during cycling. At a current density of 0.05 A·g^−1^ the Mg_x_V_5_O_12_·*n*H_2_O electrode provided a high capacity of 160 mAh·g^−1^, corresponding to 1.3 Mg^2+^ inserted per V_5_O_12_ structural unit. The Mg_x_V_5_O_12_·*n*H_2_O electrode showed excellent long-term stability (81% capacity retention after 10,000 cycles at 2 A·g^−1^), the coulombic efficiency was >99%. *In situ* XRD measurements (Figure 7e) had shown that the Mg_x_V_5_O_12_·*n*H_2_O electrode undergoes dynamic structural changes upon Mg^2+^-intercalation/deintercalation. The (001) diffraction peak shifted to a higher angle region during the discharge process (reduction), indicating the gradual contraction of the interlayer spacing. During charging (oxidation), the (001) peak returned to the initial position by the end of a deep charge. The Mg^2+^-intercalation caused a decrease in interlayer spacing to 1.10 nm, and it recovered to 1.19 nm upon Mg^2+^ deintercalation (Figure 7f,g).

Expanding the interlayer spacing of layered materials is an effective way to improve Mg^2+^-ion storage performance. On the one hand, as the interlayer spacing increases, the interaction between intercalated guest ions and host lattices weakens, which greatly improves the mobility of cations in cathode materials. On the other hand, the number of available sites in cathode materials also increases due to the enlarged lattice spaces, leading to improved reversibility of Mg^2+^-ion storage capacity.

### 2.6. Vanadium Oxides Pre-Intercalated with Organic Molecules and Conducting Polymers

The intercalation of conducting polymers into vanadium oxides has been shown to be advantageous for improving the functional properties of electrode materials. Conducting polymers intercalated into layered vanadium oxide can increase the interlayer spacing and stabilize the layered structures by reducing the coulombic interactions between the guest cations and the host framework, as has been shown on numerous examples for aqueous Zn-ion batteries [115].

**Figure 8:**
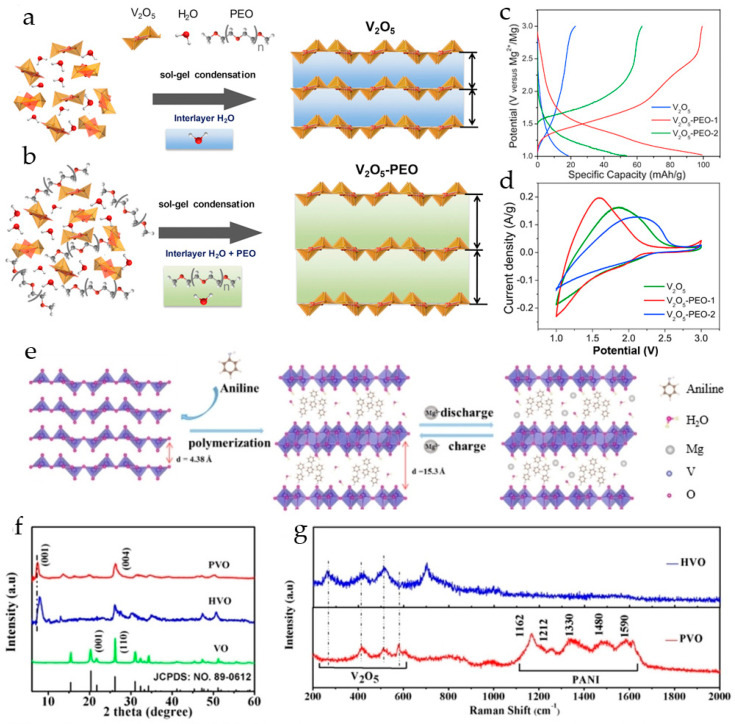
(**a**) Scheme of the growth of hydrated V_2_O_5_ nanosheets by condensation of [VO_4_]^3−^ polyanions in aqueous solution without PEO and (**b**) with PEO, (**c**) GCD profiles of V_2_O_5_, V_2_O_5_-PEO-1, and V_2_O_5_-PEO-2 at a current density of 10 mA·g^−1^, (**d**) cyclic voltammograms of V_2_O_5_, V_2_O_5_-PEO-1, and V_2_O_5_-PEO-2 at a scan rate of 2.5 mV·s^−1^. (Reprinted with permission [116]. Copyright 2017, Elsevier). (**e**) Scheme of the synthesis of the PVO superlattice, (**f**) XRD patterns of VO, HVO, and PVO, (**g**) Raman spectra of HVO and PVO. (Reprinted with permission [117]. Copyright 2021, John Wiley and Sons).

Rechargeable magnesium batteries with a V_2_O_5_ xerogel cathode with polyethylene oxide (PEO) incorporated between the oxide layers showed a significant improvement in the reversible capacity of magnesium ions [116]. PEO was introduced in the V_2_O_5_ interlayer during the V_2_O_5_ sol–gel synthesis (Figure 8a,b); this approach allowed the expansion of the interlayer spacing in V_2_O_5_ and the reduction in the interaction of intercalated divalent Mg^2+^ with the host lattice. X-ray diffraction showed that the interlayer spacing in V_2_O_5_ xerogel was increased to 12.6–13.6 Å by the inclusion of PEO. Galvanostatic charge–discharge profiles of V_2_O_5_ xerogel and V_2_O_5_-PEO nanocomposites with different amounts of PEO are shown in Figure 8c. In a cell with a Mg anode and a dry Mg(ClO_4_)_2_/AN electrolyte, the V_2_O_5_-PEO-1 nanocomposite exhibited a discharge capacity of 100.3 mAh·g^−1^ at 10 mA·g^−1^, ~5 times higher than of V_2_O_5_ xerogel (and ~2 times higher than of V_2_O_5_-PEO-2), improved stability, and enhanced rate capability. The Mg^2+^-ion capacity for the V_2_O_5_ xerogel, V_2_O_5_-PEO-1, and V_2_O_5_-PEO-2 cathode materials was determined as 0.06, 0.34, and 0.18 per V_2_O_5_ formula unit (i.e., x in Mg_x_V_2_O_5_), respectively. In cyclic voltammograms, the V_2_O_5_-PEO-1 nanocomposite showed higher currents compared to V_2_O_5_ xerogel and V_2_O_5_-PEO-2, indicating that the introduction of an optimal PEO ratio within V_2_O_5_ improved the Mg ion charge storage performance. The Mg^2+^ diffusion coefficient in V_2_O_5_-PEO-1 nanocomposite was 4.7 × 10^−11^ cm^2^·s^−1^, that is, 2 times higher than the Mg^2+^ diffusion coefficient in the V_2_O_5_ xerogel. The Mg^2+^ diffusion coefficient in V_2_O_5_-PEO-2 was 2.5 × 10^−12^ cm^2^·s^−1^, lower than that for the V_2_O_5_ xerogel. Therefore, Mg^2+^-ion diffusion was not only influenced by the interlayer spacing (which is the largest for V_2_O_5_-PEO-2), but also by the interlayer composition. It was suggested that a higher PEO ratio in the composite could result in lower charge storage for reasons such as a lower diffusion rate of Mg^2+^-ions in PEO-rich regions, lower electronic conductivity due to the insulating nature of PEO, and coordination of PEO molecules with the V_2_O_5_ lattice, blocking sites for Mg^2+^ intercalation.

PANI-V_2_O_5_ 2D organic–inorganic superlattices were synthesized and tested as cathode materials for RMBs in [117]. PANI-V_2_O_5_ (PVO) was synthesized via the intercalation of aniline monomers and subsequent interlayer polymerization (Figure 8e). In the acidic environment, the anilinium cations diffused into the interlayer space of V_2_O_5_ and underwent oxidative polymerization, V_2_O_5_ being a mild oxidizing agent. Compared to the XRD pattern of V_2_O_5_ (VO), a new diffraction peak of hydrated V_2_O_5_ (HVO) was located at 7.08°, confirming that the interlayer spacing of HVO is greater than that of VO, and for PVO, the new diffraction peak was shifted to a lower angle 6.47°, indicating larger interlayer spacing in PVO than in HVO and the insertion of PANI (Figure 8f). The formation of protonated PANI in PVO was confirmed by the Raman spectra (Figure 8g). PANI not only expanded the interlayer spacing of V_2_O_5_, but also provided additional charge storage sites. Benefitting from the above features, the PVO demonstrated high capacity (280 mAh·g^−1^ at a current density of 0.1 A·g^−1^) and excellent rate performance (135 mAh·g^−1^ at a current density of 4 A·g^−1^) in 0.3 M Mg(CF_3_SO_3_)_2_/AN electrolyte. The PVO cathode delivered capacities of 275, 250, 220, 175, 155, and 130 mAh·g^−1^ at current densities of 0.1, 0.2, 0.5, 1.0, 2.0, and 4.0 A·g^−1^, respectively. The PVO had an excellent cycling stability, and the retained capacity after 500 cycles at 4 A·g^−1^ was 80 mAh·g^−1^.

Polyaniline was *in situ* intercalated into V_6_O_13_ [118]. With the increase in the amount of aniline monomer, PANI-V_6_O_13_ composites with different enlarged *d*-spacings (11.8, 13.4, and 14.7 Å) were obtained. The contents of V_6_O_13_ and PA in the PA100-V_6_O_13_ with 13.4 Å *d*-spacing were 88.18% and 9.60%, respectively. In the cell with an activated carbon anode and a Mg(TFSI)_2_/DME electrolyte, the PA100-V_6_O_13_ sample demonstrated the highest reversible capacity (195 mAh·g^−1^ at 0.1 A·g^−1^), with a retention of 173 mAh·g^−1^ after 100 cycles. PANI-V_6_O_13_ exhibited high rate capability (46 mAh·g^−1^ at 10 A·g^−1^) and outstanding cycling stability (2500 cycles at 5 A·g^−1^). The higher reversible capacity of Mg^2+^ storage of PA100-V_6_O_13_ was mainly ascribed to the expanded interlayer spacing. The sample PA150-V_6_O_13_ with a higher polyaniline content and a larger interlayer spacing had higher initial discharge capacity but less satisfactory cycling performance, probably due to poor structural stability which reduced the capacity retention. The Mg^2+^ diffusion coefficients in PANI-V_6_O_13_ calculated from GITT data varied over the entire insertion/extraction processes and were in the range from 2.1 × 10^−9^ to 7.5 × 10^−12^ and from 2.7 × 10^−10^ to 3.2 × 10^−11^ for the insertion and extraction processes, respectively. Thus, in polyaniline-intercalated V_6_O_13_, the Mg^2+^ intercalation kinetics were enhanced due to the π-conjugated polyaniline molecules weakening strong coulombic interactions between Mg^2+^-ions and anions in the host material.

**Figure 9:**
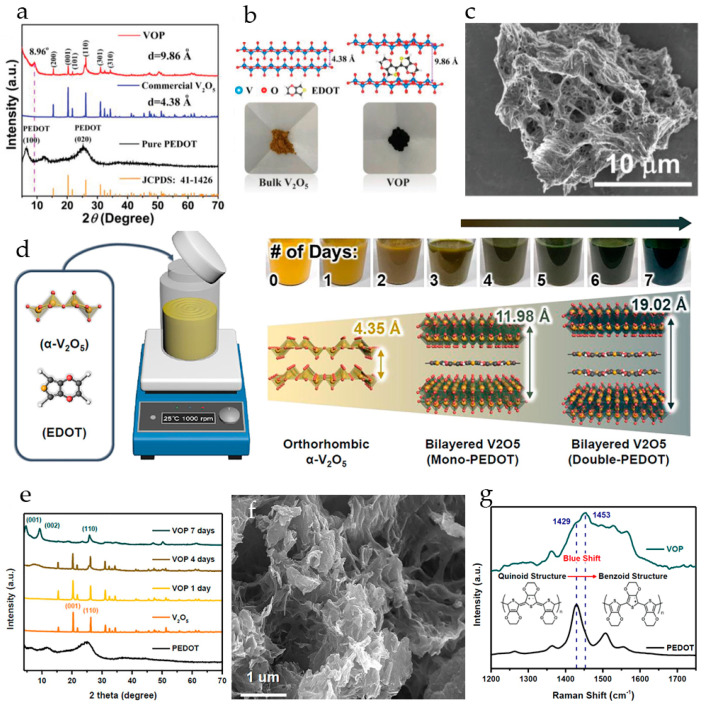
(**a**) XRD patterns of PEDOT, commercial V_2_O_5_ and as-prepared VOP composite, (**b**) schematic structure and optical images of commercial V_2_O_5_ and VOP composite, (**c**) SEM image of VOP composite. (Reprinted with permission [119]. Copyright 2021, John Wiley and Sons). (**d**) Schematic representation of the VOP synthesis, (**e**) XRD patterns of pristine V_2_O_5_, PEDOT, and as-prepared VOP, (**f**) SEM of VOP, (**g**) Raman spectra of PEDOT and blue-shifted VOP. (Reprinted with permission [120]. Copyright 2023, Elsevier).

The layered V_2_O_5_-PEDOT composite with an enlarged interlayer spacing of 9.86 Å was synthesized in [119] via *in situ* polymerization of 3,4-ethylenedioxythiophene and sequential intercalation of PEDOT and cetyltrimethylammonium bromide (CTAB) into V_2_O_5_. In the XRD pattern of the as-prepared V_2_O_5_-PEDOT (VOP), a new peak appears at 8.96°, corresponding to an increased interlayer spacing of 9.86 Å, much larger than the *d*-spacing of V_2_O_5_ (4.38 Å). The intercalation of PEDOT pillars results in dark coloring of the VOP powder; these pillars change the structure of bulk V_2_O_5_ by drastic volume expansion, resulting in delamination of the VOP into a nest-like structure of interlaced nanowires about 50 nm in diameter, as seen in the SEM image (Figure 9a–c). The magnesium storage performance of V_2_O_5_-PEDOT cathode was evaluated in a cell with a Mg anode and all-phenyl complex APC-CTAB/THF electrolyte. The CTAB was added for further improvement in the Mg^2+^ diffusion kinetics. Two pairs of broad redox peaks located at around 1.25/0.80 and 1.78/1.57 V (vs. Mg/Mg^2+^) were observed in the cyclic voltammograms of V_2_O_5_-PEDOT. The reversible capacity of V_2_O_5_-PEDOT in the full cell was 288.7 mAh·g^−1^ at 0.1 A·g^−1^, and the electrode demonstrated high cyclability (over 500 cycles at 0.5 A·g^−1^ with capacity retention 68%).

A V_2_O_5_/PEDOT (VOP) composite was synthesized by intercalating poly-3,4-ethylenedioxythiophene into V_2_O_5_ under stirring at room temperature for 1 week (Figure 9d) [120]. After 4 days of stirring, a new diffraction peak appeared in the XRD pattern of VOP at 7.6°, corresponding to an interlayer spacing of 11.98 Å. After 7 days of stirring, two new peaks appeared at 4.65° and 9.32°, corresponding to an interlayer spacing of 19.02 Å of the (001) plane and 9.49 Å of the (002) plane (Figure 9e). It was observed that the bilayer structure of orthorhombic V_2_O_5_ was retained even after the insertion of PEDOT. The porous structure of the as-prepared VOP resulting from the aggregation of the bilayer structures is shown in the SEM image (Figure 9f). The electronic coupling of PEDOT with V_2_O_5_ interlayers led to a change in PEDOT structure from the quinoid to the benzoid, and the corresponding blue shift of the principal peaks of PEDOT was observed in UV-vis spectra (Figure 9g). A reversible and fast Mg^2+^ ion insertion/extraction in/out of an enlarged interlayer spacing of 19.02 Å (in the charged state at +1.0 V) and 20.16 Å (in the discharge state at −1.0 V) was achieved. The VOP electrodes in 0.3 M Mg(TFSI)_2_/AN delivered a high specific capacity of 339.7 mAh·g^−1^ at 0.1 A·g^−1^, high rate capacity of 256.3 mAh·g^−1^ at 0.5 A·g^−1^, and long-term cyclic stability with a 0.065% decay rate and high capacity of 172.5 mAh·g^−1^ after 500 cycles. VOP delivered much higher specific capacities (320, 271, 220, 172, and 117 mAh·g^−1^) than pristine V_2_O_5_ (72.0, 67, 58, 50, and 43 mAh·g^−1^) at the current densities of 0.1, 0.2, 0.5, 1.0, and 2.0 A·g^−1^, respectively. The enhancing effect of water activation on the kinetics was confirmed. When 3 M H_2_O was added to 0.3 M Mg(TFSI)_2_/AN, the discharge capacities of VOP at 0.1 A·g^−1^ increased from 165 to 348 mAh·g^−1^, and the capacity of pristine V_2_O_5_ increased from 82 to 113 mAh·g^−1^. The improvement in the electrochemical performance of VOP and pristine V_2_O_5_ was attributed to the lowered desolvation energy of the solvated Mg^2+^-ions.

V_2_O_5_·H_2_O was synthesized and intercalated with polyacrylonitrile (PAN) by hydrothermal synthesis procedure [121]. The exact chemical composition of the PAN-intercalated oxide was V_2_O_5_·0.34H_2_O-PAN, and that of vanadium oxide used for comparison was V_2_O_5_·0.33H_2_O. According to XRD data, all peaks of the V_2_O_5_-PAN and V_2_O_5_·H_2_O were consistent with double-layer V_2_O_5_ (JCPDS No. 40-1296). The layer spacing increased from 9.91 Å in the pure V_2_O_5_·H_2_O to 10.63 Å in V_2_O_5_-PAN. It was shown that intercalation of PAN also induced cation reduction and generated abundant anion vacancies, thus improving ion diffusion and electron transfer kinetics. V_2_O_5_-PAN electrodes had perfect structural stability and electrochemical reversibility during charge–discharge. After stabilization, V_2_O_5_-PAN achieved a high specific discharge capacity of ~180 mAh·g^−1^ at 50 mA·g^−1^ in 0.5 M Mg(TFSI)_2_/AN electrolyte, and its life-span at 2 A·g^−1^ was 18,000 cycles.

The general tendency of positive effects observed with the introduction of conducting polymer molecules is explained by the formation of “pillars” expanding the interlayers, and also by the improvement in electrical conductivity and the effect of electrostatic shielding of the host from the high charge density of Mg^2+^. However, to date, the role of the composition/nature and the optimal fraction of conducting polymers introduced into the layered structure need further investigation.

## 3. Mechanisms of Intercalation and Role of Structural Water

The general operating principle of RMBs in dry aprotic electrolytes is thought to be similar to that of other metal ion batteries. It is based on the reversible intercalation/deintercalation of Mg^2+^-ions into the active material of the cathode. However, there is some controversy in the studies of the mechanism of Mg^2+^ intercalation in dry electrolytes; the reaction mechanism of α-V_2_O_5_ in RMBs with a dry electrolyte is not well understood.

A systematic study of Mg insertion into orthorhombic V_2_O_5_ was performed by combining electrochemical and structural methods [122]. The results for an electrochemically cycled V_2_O_5_ cathode in a full cell with Mg metal anode obtained by atomic-resolution transmission electron microscopy showed the local formation of the theoretically predicted ε-Mg_0.5_V_2_O_5_ phase; however, the intercalation level of Mg was low.

The investigations of the electrochemical behavior of orthorhombic α-V_2_O_5_ in dry and “wet” alkyl carbonate-based electrolytes and the insertion-driven structural changes of the active phase studied by *ex situ* X-ray diffraction showed that proton intercalation dominates the reaction of α-V_2_O_5_, even in a dry electrolyte, and the intercalation of Mg^2+^-ions is negligible [123].

*In operando* studies of the reaction mechanism of α-V_2_O_5_ cathodes in RMBs by synchrotron diffraction and *in situ* X-ray absorption near-edge spectroscopy (XANES) together with *ex situ* Raman and X-ray photoelectron spectroscopy [75] have shown the reversibility of magnesium ion intercalation and provided information on the evolution of the crystal structure and the change in oxidation degrees during charge–discharge cycling. It was shown that α-V_2_O_5_ transforms to a Mg-poor phase (Mg_0.14_V_2_O_5_) during discharging, and then undergoes a two-phase transition.

There are three related issues, the role of which in the case of vanadium oxide cathodes for RMBs should be considered in more detail:

(i) Crystalline V_2_O_5_ is capable of reversible intercalation of Mg^2+^ in “wet” organic electrolytes, containing enough water molecules to coordinate to Mg^2+^-ions and shield their high charge during intercalation, thus allowing faster diffusion of Mg^2+^-ions within the host material.
(ii) The incompatibility of metallic Mg anodes with wet electrolytes leads to the focus of the research on the bilayer V_2_O_5_, hydrated, and water-pillared vanadium oxides, where water molecules are naturally present in the lattice [104,108,111]. The interlayer water molecules effectively shield the interaction between Mg^2+^ and the host lattice, improving the reversibility of Mg^2+^-ion intercalation/deintercalation and structural stability of the cathode material during cycling.

Following the pioneering work [61], many researchers noted that V_2_O_5_ exhibits improved capacity in aprotic electrolytes containing small amounts of water. In particular, the study of the electrochemical performance of V_2_O_5_ cathodes in Mg(ClO_4_)_2_/PC and Mg(TFSI)_2_)/G2 electrolytes [124,125] showed that controllable amounts of water lead to increased specific capacities.

Water molecule intercalation into V_2_O_5_ structures improves Mg^2+^-ion diffusion kinetics due to enlarged interlayer spacing. In addition, the water molecules in the cathode increase electrostatic shielding, which is a very important factor for improving the migration rate of Mg^2+^-ions within the host material [47,124].

The experimental observations of the positive influence of water molecules in non-aqueous electrolytes on the charge storage properties of V_2_O_5_ (higher currents in CVs, higher capacities, lower polarization) were later extended by the experimental results pointing to the mechanism in which both protons and magnesium ions participate as charge carriers in the redox process.

Some works demonstrate co-intercalation of protons together with magnesium ions, providing higher capacity of vanadium oxide cathodes. A number of works demonstrate the dominant role of reversible proton insertion in the capacity of RMB in aqueous electrolytes and non-aqueous electrolytes containing small amounts of water.

The study of the influence of water additive in the organic electrolyte had shown that the presence of water enables higher capacities for an α-V_2_O_5_ cathode [125]. The capacity achieved for the wet electrolyte was ~260 mAh·g^−1^, which is close to the theoretical value. In dry electrolyte, a discharge capacity of only ~60 mAh·g^−1^ was achieved. Solid state NMR revealed that the major contribution towards the higher capacity in high water-containing electrolyte solution (1 M Mg(TFSI)_2_/G2 with 2600 ppm H_2_O) originates from the reversible proton insertion. On lowering the water level of the electrolyte (1 M Mg(TFSI)_2_/G2 with 15 ppm H_2_O), the evidence of reversible Mg intercalation was obtained by EDX of the discharged V_2_O_5_, which demonstrated the presence of a higher concentration of Mg in the discharged state compared to the charged and pristine V_2_O_5_ electrodes.

The intercalation of Mg^2+^ in α-V_2_O_5_ was again investigated in dry and water-containing organic electrolytes [126]. An improved electrochemical performance was observed after adding water to a dry organic electrolyte; this effect was explained by the co-intercalation of protons and Mg^2+^. The discharged product of α-V_2_O_5_ in a 0.5 M Mg(ClO_4_)_2_ + 2.0 M H_2_O/AN electrolyte was Mg_0.17_H_x_V_2_O_5_ (x = 0.66–1.16), indicating that the capacity is mainly due to proton intercalation.

The magnesium ion storage capability of a VO_2_(B) cathode in dry and wet 0.5 M Mg(ClO_4_)_2_/AN electrolytes was studied in [56]. The water content in dry and wet electrolytes was 40 and 650 ppm, respectively. In the dry electrolyte, VO_2_(B) deliveredan initial discharge capacity of 50.6 mAh·g^−1^ at a current density of 20 mA·g^−1^, with a coulombic efficiency of 94.4%. Under the same electrochemical test conditions, the initial discharge capacity of VO_2_(B) in the wet electrolyte was 251.0 mA·g^−1^, with a coulombic efficiency of 99.4 %. The high discharge capacity observed in the wet electrolyte was attributed to predominant proton intercalation instead of magnesium intercalation. It was also shown that VO_2_(B) may suit as a high-performance cathode material only if its particles are relatively small, to exclude long distances for Mg^2+^-ion diffusion along the diffusion channels within the material.

The strongly polarizing nature of Mg^2+^-ions leads to slow intercalation kinetics. The structural water in host electrode materials facilitates migration of Mg^2+^-ions due to the “lubricating” effect; in addition, water molecules act as charge screening media to reduce the coulombic repulsion between the ions and the host lattice.

The enhanced insertion reaction of Mg^2+^-ions into the V_2_O_5_(H_2_O)_y_ (2 < y < 3) xerogel, containing single and/or double layers of H_2_O molecules in the interlayer space of the oxide, was reported as early as 1992 in [127]. Bonded H_2_O molecules allowed smooth insertion and de-insertion of Mg^2+^-ions during the electrochemical cycling of V_2_O_5_ xerogel electrodes in a dry 1 M Mg(ClO_4_)_2_/AN electrolyte. The specific charge density 170 Ah·kg^−1^ was measured in the first cycle for V_2_O_5_ xerogel, while less than 50 Ah·kg^−1^ was observed in the first cycle for pure V_2_O_5_.

Layered H_2_V_3_O_8_ nanowires demonstrated high Mg^2+^-ion storage with a capacity of 304.2 mAh·g^−1^ at 50 mA·g^−1^ in a three-electrode cell with 0.5 M Mg(ClO_4_)_2_/AN electrolyte and activated carbon cloth as counter and reference electrodes [128]. In the cyclic voltammograms at a scan rate of 0.2 mV·s^−1^, one cathodic peak at ~2.0 V and two anodic peaks at 2.3 and 2.5 V (vs. Mg/Mg^2+^) were observed, which were ascribed to the insertion and extraction of magnesium ions. GCD curves showed that H_2_V_3_O_8_ nanowires possess a high working voltage platform of about 2.0 V. H_2_V_3_O_8_ nanowires demonstrated 85.9% capacity retention after 20 cycles at 50 mA·g^−1^.

**Figure 10:**
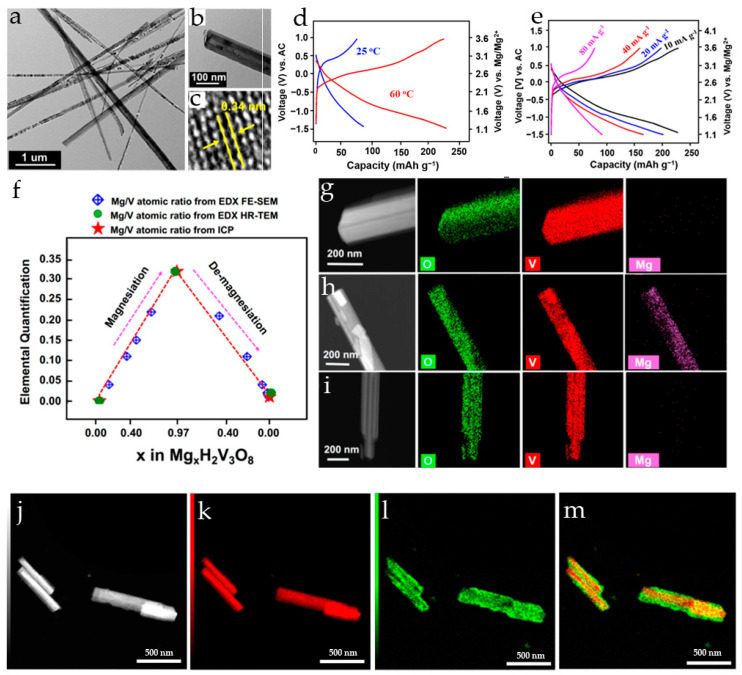
(**a**,**b**) TEM images of H_2_V_3_O_8_ nanowires, (**c**) HR-TEM image of H_2_V_3_O_8_ nanowires, (**d**) initial GCD profiles of H_2_V_3_O_8_ in 0.5 M Mg(ClO_4_)_2_/AN at 25 and 60 °C at a current density of 10 mA·g^−1^, (**e**) initial GCD profiles H_2_V_3_O_8_ at 60 °C at various current densities, (**f**) Mg/V atomic ratios for Mg_x_H_2_V_3_O_8_ electrodes during the discharge–charge cycle, (**g**) FE-TEM EDX elemental mapping of initial (x = 0) electrode, (**h**,**i**) FE-TEM EDX elemental mapping of discharged (x = 0.97), and charged (x = 0) electrodes. (Reprinted with permission from [129]. Copyright 2018, American Chemical Society). (**j**) HAADF STEM image of HVO nanofibers cycled in wet electrolyte, (**k**,**l**) corresponding V-K edge and Mg-K edge EDX intensity maps, (**m**) overlay of V-K and Mg-K edge EDX intensity maps. (Reprinted with permission from [130]. Copyright 2022, Elsevier).

Hydrated vanadium oxide H_2_V_3_O_8_ nanowires were synthesized via a one-step hydrothermal method [129]. The H_2_V_3_O_8_ nanowires had a diameter of 100–200 nm (Figure 10a,b) and an interlayer spacing of 0.34 nm (Figure 10c), corresponding to the d(011) spacing of H_2_V_3_O_8_. The material exhibited reversible magnesiation/demagnesiation with an initial discharge capacity of 80 mAh·g^−1^ and 231 mAh·g^−1^ at 10 mA·g^−1^ at 25 °C and 60 °C, respectively (Figure 10d), and an average discharge voltage of ~1.9 V (vs. Mg/Mg^2+^) in a dry (48 ppm H_2_O) 0.5 M Mg(ClO_4_)_2_/AN electrolyte. The discharge capacities of H_2_V_3_O_8_ at 60 °C were 231, 201, 170, and 97 mAh·g^−1^ at the current densities of 10, 20, 40, and 80 mA·g^−1^, respectively (Figure 10e). The capacity observed at 25 °C in dry electrolyte was much lower than the value of 304.2 mAh·g^−1^ reported for H_2_V_3_O_8_ in [128] due to the difference in the water content in the organic electrolyte. In the wet electrolyte (5790 ppm H_2_O), it increased to ~260 mAh·g^−1^. The structural water of H_2_V_3_O_8_ (V_3_O_7_·H_2_O) remained stable during cycling. The chemical formula for the discharged electrode at 60 °C was calculated as Mg_1.22_H_2_V_3_O_8_, considering that one electron charge transfer corresponds to 94.75 mAh·g^−1^ per formula unit of H_2_V_3_O_8_. The analysis of the discharged electrode suggested the chemical formula Mg_0.97_H_2_V_3_O_8_ (Figure 10f). The difference of ~20% was explained by the thorough washing of the electrodes during the sample preparation for elemental analysis, when the bulk-intercalated Mg^2+^-ions remained and the surface ions were washed out. A homogeneous distribution of Mg^2+^-ions was observed in the vanadium oxide particles of the discharged electrode, while they were absent in the initial or charged samples (Figure 10 g–i).

It was shown that the kinetic and the electrochemical properties of H_2_V_3_O_8_ (HVO) are improved by adding water to a dry organic electrolyte [130]. HVO nanofibers were synthesized by exfoliation of V_2_O_5_ followed by a hydrothermal process. H_2_V_3_O_8_ delivered an initial specific capacity of 303 mAh·g^−1^ at a current density of 50 mA·g^−1^ in a wet Mg(ClO_4_)_2_·3.1H_2_O/AN electrolyte, four times higher than in a dry electrolyte (67 mAh·g^−1^). Although the presence of water in the aprotic electrolyte was beneficial for the specific capacity, the capacity retention in the wet electrolyte was 80% after 30 cycles at 50 mA·g^−1^ and 61% after 200 cycles at 100 mA·g^−1^, while in the dry electrolyte the capacity retention was 99% after 30 cycles at 50 mA·g^−1^ and 109% after 200 cycles at 100 mA·g^−1^, demonstrating the long-term stability and the reversibility of the charge–discharge process in the dry electrolyte. In an “ambient” Mg(ClO_4_)_2_·1.4H_2_O/AN electrolyte, the HVO cathode delivered an initial specific capacity of 167 mAh·g^−1^ at a current density of 50 mA·g^−1^ and showed a capacity retention of 73% after 30 cycles, suggesting that the water content in the electrolyte affects the reversibility of the charge–discharge processes. The structural changes in HVO as a function of the water content in the electrolyte were studied by *in operando* XRD during galvanostatic cycling. It was observed that the HVO structure almost fully recovered after two discharge/charge cycles in dry electrolyte, while in the presence of water, irreversible changes in the lattice parameters occurred in the initial cycle followed by a reversible discharge/charge cycle. In the presence of water, the changes in lattice parameters and unit cell volume during discharge/charge were 8 times larger, supporting the hypothesis that H_2_O molecules from a hydration shell co-intercalate with Mg^2+^ ions, thus leading to a decrease in the cycling stability of HVO in electrolytes with water content. In the wet and “ambient” electrolytes, the insertion of ~0.60 equivalent Mg^2+^ into HVO took place. In the wet electrolyte, STEM/EDX (Figure 10 j–m) revealed that the inserted Mg^2+^-ions were mainly accumulated close to the surface and at the edges of the HVO fibers, but did not diffuse into the bulk. This explained the high reversibility of the insertion process and the small lattice parameter changes.

Introducing water molecules into the lattice material together with cation pre-intercalation is also an effective strategy for improving the electrochemical performance of V_2_O_5_ in RMBs. Mn^2+^-ions were introduced into the hydrated vanadium oxide by a hydrothermal method in [111]. The experimental results revealed the synergetic effect of both Mn^2+^ ions and crystal water in the Mn_0.04_V_2_O_5_·1.17H_2_O nanobelts, improving the structural and cycle stability of the cathode in the Mg(TFSI)_2_/AN electrolyte. The Mn_0.04_V_2_O_5_·1.17H_2_O material exhibited a high specific capacity (145 mAh·g^−1^ at 50 mA·g^−1^), good rate capability (50 mAh·g^−1^ at 4 A·g^−1^), and long cycle stability (82% capacity retention after 10,000 cycles at 2 A·g^−1^), better than that of water-free Mn_0.04_V_2_O_5._ The *in situ* and *ex situ* material characterizations revealed that the structure of Mn_0.04_V_2_O_5_·1.17H_2_O was stable during the charge–discharge process.

**Figure 11:**
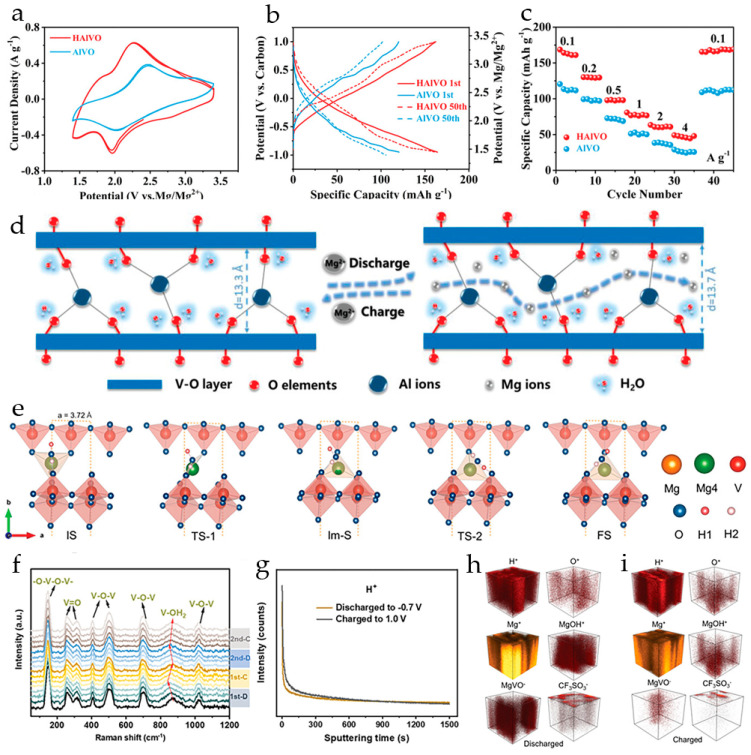
(**a**) The first two CVs of HAlVO and AlVO at the scan rate of 1 mV·s^−−1^, (**b**) the first and fiftieth charge and discharge curves of HAlVO and AlVO at the current density of 100 mA·g^−1^, (**c**) rate performance of HAlVO and AlVO at the current densities of 0.1–4.0 A·g^−1^, (**d**) schematic of the reversible insertion/extraction of Mg^2+^ ions in water-lubricated HAlVO. (Reprinted with permission from [113]. Copyright 2022, John Wiley and Sons). (**e**) Migration of Mg^2+^ ions along the *a* direction in the interlayer of MVOH during the H_2_O-Mg^2+^ waltz-like shuttle, (**f**) *in situ* Raman spectra of the MVOH cathode at different discharge/charge states, (**g**) depth profile of H^+^, (**h**,**i**) TOF-SIMS imaging of H^+^, O^+^, Mg^+^, MgOH^+^, MgVO^−^, and CF_3_SO_3_^−^ in fully discharged and charged electrode [107].

Water-lubricated and aluminum ion-pillared vanadate H_11_Al_2_V_6_O_23.2_ (HAlVO) was reported as a high-performance cathode material for Mg^2+^ ion storage [113]. The capacity fade mechanism of water-free aluminum vanadate AlV_3_O_9_ (AlVO) was also investigated. The charge transfer process in water-lubricated and water-free aluminum vanadates was analyzed by DFT calculations, and the different charge transfer processes in the two materials and the charge shielding effect of the water molecule in HAlVO were revealed. The comparison of the cyclic voltammograms of HAlVO and AlVO showed that the shape of the CVs and the position of the redox peaks of the two materials are different: HAlVO had more intense reduction and oxidation peaks than AlVO (Figure 11a). HAlVO displayed higher initial specific capacity and a more stable cycle performance (165 mAh·g^−1^ in the 1st and 50th cycles), while AlVO exhibited a capacity of only 125 mAh·g^−1^ in the 1st cycle, which decreased to 105 mAh·g^−1^ in the 50th cycle (Figure 11b,c). Water-lubricated HAlVO had better cycle performance at all current densities. The diffusion coefficients were obtained for HAlVO and AlVO from GITT curves at different stages of Mg^2+^ insertion. At the initial stage of Mg^2+^ insertion, *D*_HAlVO_ was 4.45 × 10^−11^ cm^2^·s^−1^ and *D*_AlVO_ was 2.77 × 10^−11^ cm^2^·s^−1^. When the amount of inserted Mg^2+^ reached 100%, *D*_HAlVO_ decreased to 1.01 × 10^−11^ cm^2^·s^−1^ and *D*_AlVO_ to 2.12 × 10^−12^ cm^2^·s^−1^. The faster decrease in the diffusion rate of Mg^2+^ in AlVO than in HAlVO was explained by larger interlayer spacing of HAlVO and the shielding effect of interlayer water molecules, providing more active storage sites for Mg^2+^, enough diffusion space, and less charge repulsion when a large amount of Mg^2+^-ions is intercalated into HAlVO. The scheme of reversible insertion/extraction of Mg^2+^ in HAlVO is shown in Figure 11d. Pre-intercalated Al^3+^ ions act as “pillars” which stabilize the V-O layered structure both in discharged and charge states, which improves the cyclic stability of the electrode. When Mg^2+^ ions are inserted, the water molecules in the interlayer space facilitate achieving local electroneutrality and a lower Mg diffusion barrier; this improves the rate performance of HAlVO. The DFT calculation results suggested that the water molecules also reduce the insertion energy barrier of Mg^2+^, thus improving the specific capacity of HAlVO. The charge transfer occurs mainly from the Mg^2+^ to O of the water molecule, and less from Mg^2+^ to O in the V-O layer, thus probably preventing the structural decay of the cathode during cycling.

A Mg^2+^ pre-intercalated hydrated V_10_O_24_·nH_2_O layered material was obtained [107]. Mg_0.75_V_10_O_24_·4H_2_O (MVOH) had an interlayer spacing of 13.9 Å, much larger than that of the hydrated Mg_x_V_5_O_12_ (11.9 Å) reported in [108]. The nanoflower morphology greatly increased the specific surface area of the MVOH (33.07 m^2^·g^−1^) and provided short diffusion paths for Mg^2+^ from bulk solution to active sites, accelerating the insertion/extraction of Mg^2+^. The diffusion coefficient of Mg^2+^ in the MVOH calculated from the EIS data was as high as 1.08 × 10^−9^ cm^2^·s^−1^, indicating fast migration kinetics inside the MVOH crystal lattice. In a three-electrode cell with a platinum counter electrode, a saturated calomel reference electrode (SCE), and 2 M Mg(CF_3_SO_3_)_2_ aqueous electrolyte, the MVOH cathode exhibited a high discharge capacity of 350 mAh·g^−1^ (at 0.05 A·g^−1^) and 70 mAh·g^−1^ at a higher current density of 4 A·g^−1^. The full cell with a MVOH cathode, perylene-3,4,9,10-tetracarboxylic dianhydride (PTCDA) as an anode, and 2 M Mg(CF_3_SO_3_)_2_ in PEG/H_2_O (1:1) electrolyte, delivered a discharge capacity of 133 mAh·g^−1^ at a current density of 0.02 A·g^−1^ and 42 mAh·g^−1^ at a high current density of 4 A·g^−1^, demonstrating high rate capability and satisfactory cycling stability with a capacity retention of 62% after 5000 cycles at 4 A·g^−1^. DFT calculations performed on the basis of structural characterization results demonstrated that pre-intercalated Mg^2+^-cations and structural H_2_O molecules stabilize the layered structure of MVOH. Based on the determined pre-intercalated structure of MVOH, DFT calculations were performed to identify the energetically favorable sites for Mg^2+^ to occupy and the nearest neighboring site for Mg^2+^ to hop to. Based on the relaxed structures, the waltz-like like shuttle mechanism of H_2_O molecules coordinated with Mg^2+^ transport was demonstrated during the charge–discharge of the MVOH cathode. It was shown that the Mg^2+^-ion migrates together with the coordinated water molecules, which adjust their orientation by rotating or flipping to facilitate the zig-zag migration of Mg^2+^-ions (Figure 11e). After the extraction of Mg^2+^ from MVOH, the Mg^2+^-H_2_O bonds break, and the lattice H_2_O molecules rearrange and form H-bonds in the interlayer of MVOH to stabilize the lamellar structure. To verify the Mg^2+^ shuttling of lattice H_2_O molecules, the MVOH cathode was investigated by *in situ* Raman spectroscopy during discharging/charging, and a variation in the peak at 871 cm^−1^, corresponding to the V-OH_2_ bonds, at different discharged/charged states, was observed (Figure 11f). To further verify the Mg^2+^ shuttle function of lattice H_2_O molecules, the fully discharged and charged MVOH cathode was analyzed by time of flight–secondary ion mass spectrometry (TOF-SIMS). The much higher intensity of the MgVO^−^ signal in the discharged MVOH than in the charged MVOH indicates successful insertion of Mg^2+^ during discharge, and the higher MgOH^+^ signal in the discharged MVOH than in the charged MVOH indicates strong coordination of lattice H_2_O molecules with Mg^2+^-ions inserted during discharge, confirming the H_2_O–Mg^2+^ shuttle mechanism. The same intensity and distribution of the H^+^ signal in discharged and charged MVOH indicate that the H_2_O-Mg^2+^ waltz-like shuttle mechanism eliminates the co-insertion or co-extraction of H^+^ together with Mg^2+^ (Figure 11g–i).

To sum up, the investigations of vanadium oxide-based cathodes in RMBs show that the water content, even in trace amounts, has an important influence on the mechanism of intercalation processes in organic electrolytes. Although the exact mechanism of the effect of the water molecules present in organic electrolytes on the increase in the specific capacity of vanadium oxide-based cathodes is not fully understood, it has been emphasized by many researchers. The following explanations have been proposed:

(i) Water molecules can solvate the magnesium ions and shield their divalent charge to facilitate insertion into the interlayer spaces of VO-based cathodes.
(ii) Water molecules inserted together with magnesium ions solvate the interstitial space and inner -V=O groups, and can lubricate Mg^2+^ diffusion.
(iii) Insertion or co-insertion of protons (from water molecules) takes place.
(iv) Water molecules can react with the surface -V=O groups to form stable structural hydroxyl groups to reduce the migration barrier of magnesium insertion.

A better understanding of these effects is also necessary for the proper design of compatible cathodes and electrolytes.

For more illustrative comparison, the main electrochemical characteristics of selected vanadium oxide-based cathode materials are listed in Table 2.

## 4. Conclusions

RMBs are probably one of the most promising next-generation energy storage technologies due to their high theoretical energy density, high safety, abundant resources, and low cost. The design of novel cathode materials for RMBs is the focus of investigations for the future development of RMBs.

Vanadium oxide-based cathode materials present many advantages, including low cost, high safety, and great opportunity to tune the ion transport channels, which resulted in their improved electrochemical performance in RMBs.

Although great progress has been made in the development of vanadium oxide-based cathodes for RMBs, a number of challenges remain to be overcome in the future. The main challenge in the development of cathodes for RMBs is the sluggish kinetics of Mg^2+^-ion intercalation and diffusion in electrode materials due to the strong interaction between doubly charged Mg^2+^-ions and vanadium oxide host materials.

For the development of new cathodes with improved electrochemical performance, different synthetic procedures that allow new morphological modifications of vanadium oxide-based materials, pre-intercalation of metal ions and organic (mono- and polymeric) molecules into the interlayer space can be proposed. Pre-intercalated species act as pillars between the layers, stabilize the crystal structure of the material, and prevent amorphization and dissolution of oxides during repeated insertion/extraction of Mg^2+^-ions. A pre-intercalation strategy allows the acquisition of materials with increased reversibility of Mg^2+^ (de)intercalation processes and increased interlayer/channel distances, which significantly improve the Mg^2+^-ion diffusion kinetics and possibly reduce the desolvation barriers. Deeper understanding of the effects of different pre-intercalation strategies, their optimization, and their combination with other approaches will facilitate the achievement of high-performance RMB cathodes. Further searching for possible modifiers that act as pillars represents a perspective means of cathode improvement.

Modification of vanadium oxides with highly conductive materials, such as nanocarbons, and especially conducting polymers, is also a promising means of enhancing the intrinsic conductivity of vanadium-based electrode materials and facilitating Mg^2+^ migration.

Excessive removal of interlayer water results in the loss of capacity of highly crystalline vanadium oxide structures. Mild annealing of vanadium oxides during synthesis should be considered to preserve an adequate amount of crystal water necessary to lubricate the interlayer space and weaken an interaction between oxygen sub-lattices and Mg^2+^ ions, providing electrostatic shielding. In combination with pre-intercalated metal ions or organic molecules, interlayer water also plays an important role in maintaining sufficient interlayer space. The role that traces of water in the organic electrolytes play in facilitating Mg^2+^ ion insertion also needs to be elucidated for further design of high-performance RMBs.

In addition to designing cathode materials with improved electrochemical performance, electrode/electrolyte interphase control is an important issue for stable electrochemical performance in RMBs. More attention should also be paid to the development of electrolytes compatible with cathodes and anodes.

## Figures and Tables

**Table 2:** Summary of electrochemical performance of selected vanadium oxide-based cathodes in RMBs.

Cathode Material	Synthesis Method	Morphology/ Interlayer Spacing, Å	Electrolyte	Specific Capacity, mAh·g^−1^ (Current Density, A·g^−1^)	Capacity Retention (Number of Cycles and Current, A·g^−1^)	Ref.
**V_2_O_5_** **thin film**	vacuum deposition	200 nm film	0.5 M Mg(ClO_4_)_2_/AN	180 (0.15 mV·s^−1^)	83% (36, 0.15 mV·s^−1^)	[71]
** α-V_2_O_5_**	commercial product	powder	1 M Mg(TFSI)_2_/G2, 15 ppm H_2_O	80 (20 μA·cm^−2^), initial 60 (20 μA·cm^−2^)	94% (2–10, 0.05)	[125]
** α-V_2_O_5_**	commercial product	powder	1 M Mg(TFSI)_2_/G2, 2600 ppm H_2_O	250 (20 μA·cm^−2^)	-	[125]
** α-V_2_O_5_**	commercial product	powder	0.5 M Mg(TFSI)_2_/PY_14_TFSI, at 110 °C	295 (C/5)	83.8% (50, C/5)	[72]
** α-V_2_O_5_**	precipitation, calcination	spheres/11.52	0.2 M Mg(ClO_4_)_2_/AN	225.0 (0.01), initial 190 (0.01) 100 (0.03) 55 (0.05) 40 (0.08)	95% (50, 0.05) 87% (100, 0.05)	[77]
** V_2_O_5_ film **	AACVD	thin film	0.075 M MgCl_2_	427(5.9) 170 (2.4)	82% (2000, 5.9)	[73]
** V_2_O_5_·*n*H_2_O **	ion-exchange	bilayered	1 M Mg(TFSI)_2_/G2	50 (20 µA·cm^−2^)	100% (10, 20 µA·cm^−2^)	[92]
** V_2_O_5_·*n*H_2_O **	hydrothermal method	bilayered, 2D nanopapers/10.6	2 M Mg(CF_3_SO_3_)_2_/H_2_O	162.8 (0.2) 128 (0.3) 116.7 (0.5) 104.4 (1.0) 100.4 (2.0) 91.9 (4.0) 86 (6.0) 81.3 (10.0)	70.1% (70, 0.2) 88% (1000, 10.0)	[93]
**VOx-NT**	hydrothermal	nanotubes	0.25 M Mg(AlBu_2_Cl_2_)_2_/THF	75 (0.005)	-	[131]
**VO_x_-NT**	microwave- assisted hydrothermal process	nanotubes	0.5 M Mg(ClO4)_2_/AN	218 (0.06)	70.8% (20, 0.06)	[81]
**VO_x_-NT**	microwave- assisted hydrothermal process	open-ended multilayered nanotubes	0.5 M Mg(ClO_4_)_2_/TMS-EA	124.0 (0.06)	50% (80, 0.06)	[82]
**V_2_O_5_**	hydrothermal synthesis	3 μm microspheres/25 nm thick nanosheets	0.25 M Mg(AlCl_2_EtBu)_2_/THF	126.2 (0.05), initial 92 (0.1) 80 (0.15) 62 (0.2)	71.9% (80, 0.05)	[76]
**V_5_O_12_·6H_2_O**	hydrothermal synthesis	nanoflowers	0.8 M PhMgCl + 0.4 M AlCl_3_/THF	234.3 (0.01) 53.2 (0.5)	82.1% (400, 0.05) 74.5% (1500, 0.1)	[78]
**V_6_O_13_**	Microwave- assisted	2D nanosheets/monoclinic	1.0 M Mg(ClO_4_)_2_/AN	324.0 (0.02) 278.0 (0.04) 244.0 (0.06) 214.0 (0.08)	71.9% (30, 0.04)	[79]
**VO_2_ (B)**	hydrothermal process	nanorods/12.093	1.0 M Mg(ClO_4_)_2_/AN, wet	391.0 (0.025) 370 (0.05) 341 (0.1)	94.7% (10, 0.025)	[55]
**V_2_O_5_** nanoclusters on porous carbon frameworks	ambient hydrolysis deposition	amorphous (XRD)/orthorhombic (^51^V NMR)	0.2 M [Mg_2_(μ-Cl)_2_(DME)_4_][AlCl_4_]_2_/DME	350 (0.04), initial 225 (0.04) 180 (0.08) 120 (0.320) 100 (0.640)	75% (50, 0.04) 67% (100, 0.320)	[74]
**V_2_O_5_/GO**	Solvothermal process	microparticles, /11.512	0.25 M Mg(AlCl_2_EtBu)_2_/THF	178 (0.2C), initial	78.6% (20, 0.2C)	[97]
** V_2_O_5_·1.42 H_2_O@rGO **	melt quenching process and freeze drying	nanowires /~11.3	0.5 M Mg(TFSI)_2_/AN	320 (0.05) 280 (0.1) ~100 (2.0)	81% (200, 1.0)	[98]
** V_2_O_5_·0.4H_2_O/C **	evaporation of solution	xerogel, bilayer	4.7 M Mg(NO_3_)_2_	88 (1.0), initial 102 (1.0), reversible 62 (2.0) 53 (3.0) 47 (4.0) 44 (5.0)	82.7% (10, 1.0)	[89]
** V_2_O_3_@GO **	hydrothermal/spray-drying/annealing in Ar	3D graphene- supported nanoparticles	0.3 M Mg(TFSI)_2_/AN	291.3 (0.05) 280.1 (0.1) 259.1 (0.2) 232.5 (0.5) 210.4 (1.0) 185.3 (2.0)	88.5% (1000, 0.5)	[99]
**V_2_O_3_@C**	hydrothermal/annealing in air/carbonization	mesoporous nanorods	0.3 M Mg(TFSI)_2_/AN (H_2_O)	381.0 (0.2) 354.8 (0.5) 292.7 (5.0) 208.0 (20.0) 130.4 (50.0)	60.0% (1000, 0.5)	[100]
**V_2_O_5_-PEDOT**	chemical intercalation/polymerization	/9.86	0.4 M APC/THF + 0.2 M CTAB	288.7 (0.1) 153.8 (0.3) 110.7 (0.5) in full cell	58% (100, 0.1) ~68% (500, 0.5)	[119]
**V_2_O_5_/PEDOT**	chemical intercalation/polymerization	porous, bilayered/ 19.02	0.3 M Mg(TFSI)_2_/AN + 3M H_2_O	339.7 (0.1), initial 256.3 (0.5), initial 320.4 (0.1), reversible 271.2 (0.2) 219.6 (0.5) 172.3 (1.0) 117.5 (2.0)	60.85% (200, 0.1) 67.3% (500, 0.5)	[120]
**V_6_O_13 -_PANI**	solvothermal, *in situ* intercalation	nanobelts /13.4	0.5 M Mg(TFSI)_2_/DME	195 (0.1), initial 168 (0.1), 147 (0.2) 127 (0.5) 99 (1.0) 65 (5.0) 46 (10.0)	89% (100, 0.1) 100% (2500, 5.0)	[118]
** PANI-V_2_O_5_** ** (V_2_O_5 _** **⋅** **_ _** ** 0.35C_6_H_6_N ** ** ** **⋅** ** ** ** 0.92H_2_O) **	intercalation of monomers and subsequent interlayer polymerization	2D organic- inorganic superlattices /15.3	0.3 M Mg(TFSI)_2_/AN, 0.2 M Mg(CF_3_SO_3_)_2_-MgCl_2_-AlCl_3_/DME (full cell)	280 (0.1), initial 275 (0.1), reversible 250 (0.2) 220 (0.5) 175 (1.0) 155 (2.0) 130 (4.0) 115 (0.1), full cell	70% (500, 4.0) 69.5% (50, 0.1) (full cell)	[117]
** V_2_O_5_· ** ** 0.34H_2_O·PAN **	hydrothermal synthesis	/10.63	0.5 M Mg(TFSI)_2_/AN	~180 (0.05), initial 143.2 (0.05) ~75 (0.5) 120.6 (0.1) (full cell, initial)	91% (18,000, 2.0) 41.5% (80, 0.1), full cell	[121]
** PEO-V_2_O_5_**	sol-gel synthesis	nanosheets /12.6	0.5 M Mg(ClO_4_)_2_/AN	125 (0.01), initial 100.3 (0.01)	77% (35, 0.01)	[116]
** H_2_V_3_O_8_**	hydrothermal process	nanowires /0.467 nm	0.5 M Mg(ClO_4_)_2_/AN	304.2 (0.05)	89.5% (20, 0.05)	[128]
** H_2_V_3_O_8_**	hydrothermal process	nanowires /0.34 nm	0.5 M Mg(ClO_4_)_2_/AN 60 °C	231 (0.01) 201 (0.02) 170 (0.04) 97 (0.08)	95.2% (30, 0.01)	[129]
** H_2_V_3_O_8_**	hydrothermal process	nanowires	Mg(ClO_4_)_2_· 3.1 H_2_O/AN	303 (0.05)	80% (30, 0.05) 61% (200, 0.1)	[130]
** H_2_V_3_O_8_**	hydrothermal process	nanowires	0.25 M Mg(ClO_4_)_2_/AN	67 (0.05)	99% (30, 0.05) 109% (200, 0.1)	[130]
**NaV_6_O_15_**	hydrothermal process, annealed in air	rod-like, monoclinic /10.08	0.5 M Mg(ClO_4_)_2_/AN	213.4 (0.01), initial 210.1 (0.01) 137.0 (0.02) 111.7 (0.05) 80.2 (0.1) 52.3 (0.2) 27.2 (0.5)	87% (100, 0.02)	[103]
** Na_2_V_6_O_16_· ** ** 1.63 H_2_O **	hydrothermal process	nanowires /7.9	0.5 M Mg(TFSI)_2_/DME	228 (0.02) 193 (0.03) 175 (0.05) 105 (0.08) 91 (0.1) 65 (0.2)	71% (450, 0.2)	[104]
**NaV_3_O_8_**	hydrothermal method, annealed in air	nanowires /6.85	0.5 M Mg(ClO_4_)_2_/AN	260.0 (0.05) 184.0 (0.1) 123.9 (0.2) 86.6 (0.5) 62.4 (1.0)	85.8% (30, 0.1) 88.3% (100, 0.5)	[101]
**NaV_3_O_8_·** **1.69H_2_O**	solvothermal at RT	nanobelts	0.4 M (PhMgCl)_2_-AlCl_3_/THF	150.0 (0.01), initial 110.0 (0.01), 5 cycles 55.0 (0.02)	80% (100, 0.08)	[102]
**NH_4_V_4_O_10_**	hydrothermal method	nanosheets/11.57	0.5 M Mg(ClO_4_)_2_/AN	175 (0.2 C) 128 (0.5 C) 110 (1.0 C) 69 (2.0 C)	36.5% (100, 1C)	[106].
** Zr-NH_4_V_4_O_10_**	hydrothermal method	nanorods/11.572	0.5 M Mg(ClO_4_)_2_/AN	328 (0.04) initial 300 (0.04) 240 (0.1) 207 (0.2) 175 (0.4) 147 (0.8) 100 (0.01), full cell	86% (150, 0.04)	[105]
**Mg_0.3_V_2_O_5_·** **1.1H_2_O**	solvothermal	nanowires (150 nm)/11.9	0.3 M Mg(TFSI)_2_/AN	162 (0.1) 145 (0.2) 134 (0.5) 120 (1.0) 85 (2.0) 50 (4.0)	~100% (10,000, 1.0) 80% (10,000, 2.0)	[110]
**Mg_0.1_V_2_O_5_·** **1.8H_2_O**	sol-gel method	agglomerates of sub-micron sized particles/ 12.3	0.5 M Mg(TFSI)_2_/AN	300 (C/10), initial 250–280 (C/10)	93% (7, C/10)	[109]
**Mg_x_V_5_O_12_·** **nH_2_O**	solvothermal at near ambient conditions	nanofibers/ 11.9	0.3 M Mg(TFSI)_2_/AN	~160 (0.05)	89% (300, 0.05) 81% (10,000, 2.0)	[108]
**Mg_0.75_V_10_O_24_·** **nH_2_O**	one-step precipitation method	nanoflowers/13.9	2 M Mg(CF_3_SO_3_)2/H_2_O	350 (0.05) 149 (1.0) 70 (4.0)	64% (100, 3.0)	[107]
** Mn_0.04_V_2_O_5_· ** ** 1.17H_2_O **	hydrothermal method	nanobelts/ 10.9	0.3 M Mg(TFSI)_2_/AN	145 (0.05) 131 (0.1) 110 (0.2) 108 (0.5) 97 (1.0) 80 (2.0) 50 (4.0)	82% (10,000, 2.0)	[111]
**H_11_Al_2_V_6_O_23.2_**	Hydrothermal method	Urchin-like microspheres/ 13.3	0.3 M Mg(TFSI)_2_/AN	165 (0.1) 127 (0.2) 98 (0.5) 75 (1.0) 60 (2.0) 47 (4.0)	98% (50, 0.1) 87% (3000, 1.0)	[113]

## Data Availability

There are no research data to share.

## Abbreviations

AN	acetonitrile
APC	all-phenyl complex electrolyte/phenylmagnesium chloride (PhMgCl + AlCl_3_)
CTAB	cetyltrimethylammonium bromide
DME	dimethoxyethane
EA	ethyl acetate
G2	diglyme
PC	propylenecarbonate
PEDOT	poly(3,4-ethylenedioxythiophene)
PY_14_TFSI	1-butyl-1-methylpyrrolidinium bis(trifluoromethylsulfonyl)imide
TFSI	trifluoromethylsulfonylimide
THF	tetrahydrofurane
TMS	tetramethylsilane
